# Aggregation-Induced Emission Enhancement and Solid-State Photoswitching of Crystalline Carbazole *N*-Salicylidene Anilines

**DOI:** 10.1021/acsomega.4c04764

**Published:** 2024-08-23

**Authors:** Dazaet Galicia-Badillo, José L. Belmonte-Vázquez, Mario Rodríguez, Braulio Rodríguez-Molina, Ma. Carmen García-González

**Affiliations:** † Instituto de Química (IQ), Universidad Nacional Autónoma de México (UNAM), Circuito Exterior s/n, Ciudad Universitaria, Coyoacán, Ciudad de México 04510, México; ‡ Departamento de Química Orgánica, Facultad de Química (FQ), Universidad Nacional Autónoma de México (UNAM), Ciudad Universitaria, Ciudad de México 04510, México; § Research Group of Optical Properties of Materials (GPOM), Centro de Investigaciones en Óptica, CIO, A.P. 1-948, León, Guanajuato 37000, México

## Abstract

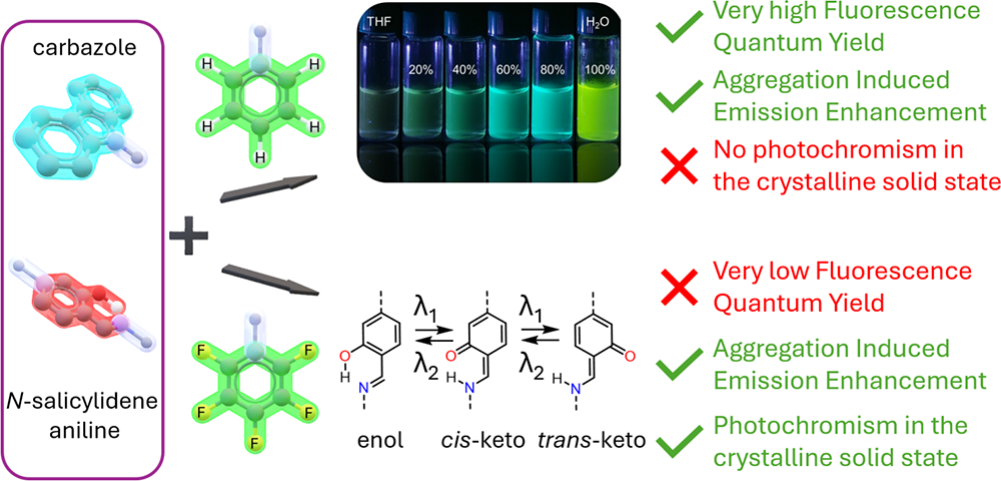

The development of fluorescent stimuli-responsive organic materials has attracted substantial interest due to their increasing optoelectronic applications. This study systematically introduces fluorine atoms on one end of carbazole-based *N*-salicylidene anilines **5a**–**5f** to elucidate the impact in their solution and solid-state photophysics. The addition of fluorine atoms at one end of the molecule induced significant changes, for example, a reduction in the quantum yield (QY) fluorescence emission in solution, going from QY near unity in compound **5a** (QY ∼ 100%) to a negligible emission in **5f** (QY < 1%). Similarly, compound **5a** showed a very strong aggregation-induced enhancement emission behavior, whereas compounds with a higher fluorine content were almost quenched. Furthermore, the crystalline solid-state photoisomerization in *N*-salicylidene anilines is not trivial, and only compounds with three (**5e**) and five fluorine atoms (**5f**) exhibited reversible solid-state photoisomerization under 405 nm light source irradiation. We propose that the presence of the arene-perfluoroarene interaction in the crystalline array facilitates the latter behavior. Our findings present a comprehensive study of crystal engineering for the obtention of photoswitchable crystalline materials and adjustable photophysics response, paving the way for its implementation in other systems.

## Introduction

The design and synthesis of pure organic luminescent materials are the subject of intense research given their numerous optoelectronic applications, including light-emitting diodes,^1^ organic field-effect transistors,^2^ chemical sensors,^3^ materials for bioimaging,^4^ or stimuli-responsive materials.^5^ To this end, continuous efforts are directed toward developing simple yet functional turn-on fluorescent materials, such as compounds based on a Schiff-base moiety.^6,7^ The materials based on that structure offer several advantages, such as low toxicity or relatively direct synthetic methods. They can also be structurally modified with a wide range of functional groups, making them ideal candidates for the development of new smart materials.^8^

*N*-Salicylidene anilines are well-known photoactive molecules that exhibit various phenomena in both solution and the crystalline solid state, such as thermochromism,^11^ or photochromism,^9^ or a combination of both.^10^ Thermochromism is attributed to modifications in ground state keto−enol tautomerism equilibria,^11^ and photochromism can be due to excited state intramolecular proton transfer^12^ or photoisomerization.^13^ It has been suggested that the “dihedral angle rule” dictates whether a compound exhibits a photochromic response. However, this rule states that the dihedral angle primarily influences the intensity of the photochromic effect rather than determining its existence.^14^ Regulating the balance between thermochromism and photochromism in the crystalline solid state is still very challenging. Some strategies to control their responsive behavior have been developed, such as cocrystallization^15^ or incorporation in metal–organic frameworks as linkers^16^ or guests^17^ with applications in various fields, including molecular photoswitching^18,19^ or data storage.^20^

Achieving densely packed photochromic crystals remains an exciting challenge, mainly when using common groups such as Schiff bases that require significant changes in volume upon isomerization within crystals. To address this issue, bulky surrounding groups may facilitate the isomerization process, thus increasing the probability of the phenomenon occurring in the crystalline solid state.^21^

**Figure 1:**
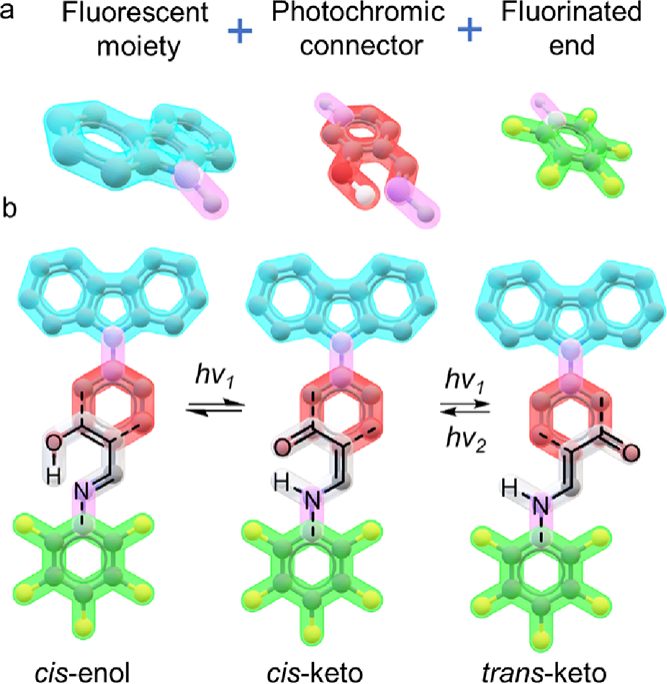
(a) Components of the carbazole *N*-salicylidene anilines reported in this work. (b) Different envisioned states for compound **5f** as a photoswitch.

Considering the reported photochromic behavior of *N*-salicylidene anilines,^22^ in this work, we explored the synthesis of Schiff bases with progressively fluorinated phenyl rings at one end. The *N*-salicylidene anilines were linked to a carbazole component to explore their resulting emissive behavior (Figure 1a). Fluorinated derivatives have been reported to change the supramolecular array through new noncovalent interactions. In some cases, perfluorination may significantly improve the mechanical strength of nanotubes,^23^ the change of the photophysical properties in solution,^24^ or the modulation of the fluorescence in cocrystals.^25^ We envisioned that adding an increasing number of halogen atoms in the periphery of the aniline precursor of the Schiff base might result in (1) changes in the fluorescence response in solution as in aggregates due to the electron-withdrawing nature of the fluorine atoms and (2) changes in the crystalline array perhaps leading to solid-state photochromism (Figure 1b). We have found that all compounds are aggregation-induced emission enhancement luminogens (AIEEgens).^26^ Furthermore, such behavior is more pronounced in compound **5a** with no fluorine atoms, showing an excellent solid-state quantum yield. Conversely, we switch from a nonphotochromic crystal, **5a**, to two fluorinated crystals, **5e** and **5f**, with reversible photochromism, possibly due to the arene-perfluoroarene interactions in their crystal packing.

## Experimental Section

Synthesis of all compounds was carried out in a two-step methodology with a C–N Ullmann reaction followed by an imine condensation. All the compounds were crystallized by slow evaporation from CH_2_Cl_2_ and structurally characterized by single crystal X-ray diffraction. Detailed crystallographic analysis was carried out with Crystal Maker and Mercury software. Dissolution steady-state absorption and emission spectra were recorded with a concentration of 4 μM in different dissolvents, with every sample excited at the absorption maxima; for aggregation experiments, the media were gradually changed from 100% THF to 100% water. The luminescence lifetime was measured using the standard time-correlated single-photon counting technique using a picosecond pulsed diode laser of wavelength 405 nm from Edinburg Instruments (EPL-405). For photoisomerization experiments, a diode laser at 405 nm (57 mW) was employed. DFT or TDDFT calculations were carried out with the CAM-B3LYP functional and the 6-311++g(d,p) basis set. The detailed experimental section can be found in the Supporting Information.

## Results and Discussion

### Synthesis of Carbazole-*N*-salicylidene Anilines

**Scheme 1:**
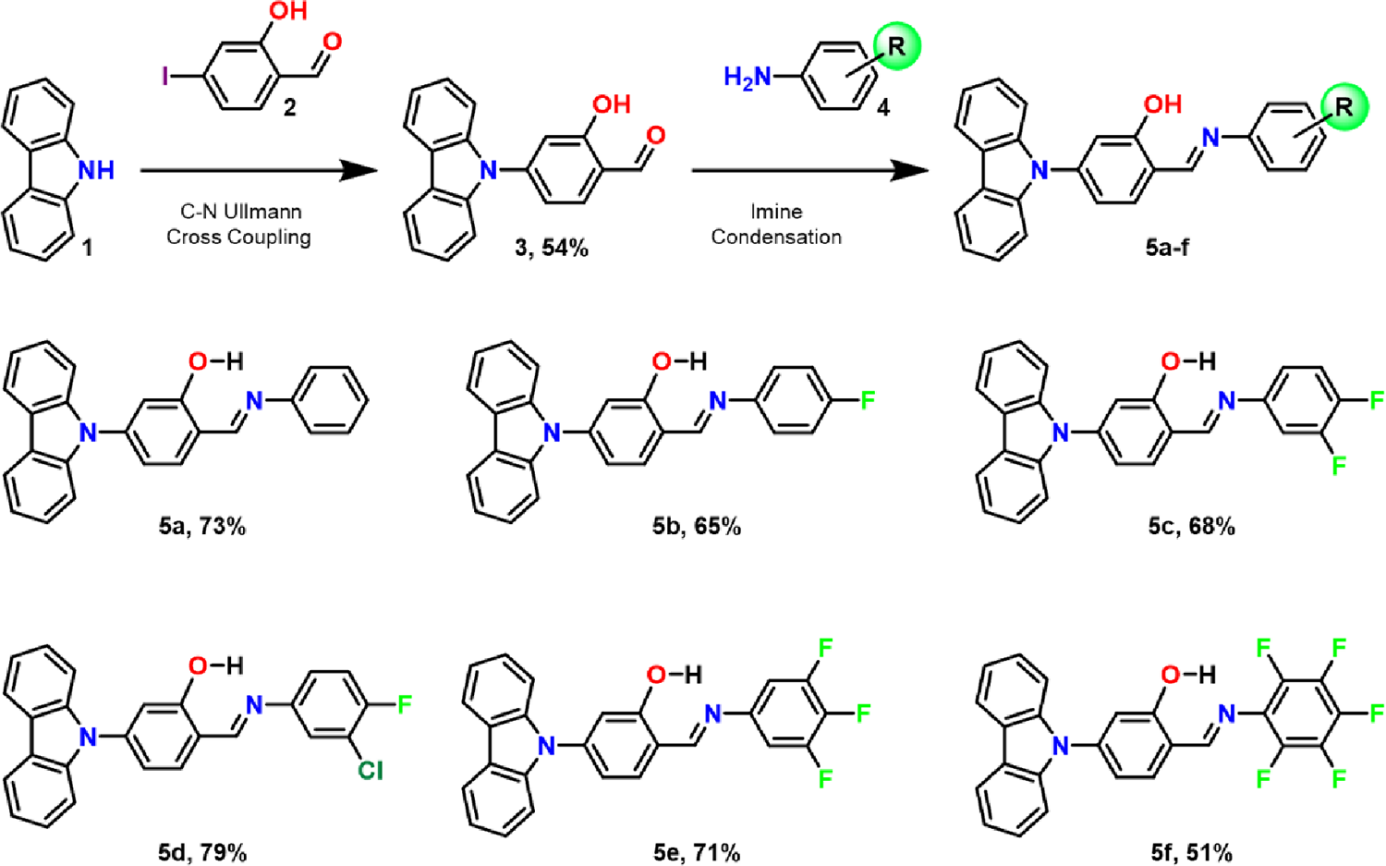
Synthesis of Carbazole-*N*-salicylidene Anilines through a Two-Step Methodology Involving an Ullmann C–N Coupling Followed by a Condensation

The preparation of compounds **5a**–**5f** was carried out by performing a C–N Ullmann-type coupling reaction between compounds **1** and **2**, as outlined in Scheme 1. To this end, compound **2** was synthesized according to the literature protocol.^27^ An Ullmann reaction with commercially available carbazole was used to afford derivative **3** with a 26% yield. Better results were achieved for the obtention of compound **3** when the reaction was conducted using microwave irradiation (100 W for 2 h at 150 °C), affording 54% yield. We also included a chlorine atom in **5d**. However, its crystalline array is isostructural with compound **5c** having two fluorine atoms. The −Cl atom does not play a significant role in the emissive properties. Subsequently, the condensation reaction between **3** and different substituted anilines **4a**–**4f** produced *N*-salicylidene anilines **5a**–**5f** with yields ranging from 51 to 79% (Scheme 1). All of the compounds were characterized by ^1^H and ^13^C solution NMR, FTIR, and high-resolution mass spectrometry (Section S2).

### Structural Characterization by Single-Crystal X-ray Diffraction

All synthesized compounds were crystallized by slow evaporation from CH_2_Cl_2_, and suitable specimens were studied by using single-crystal X-ray diffraction. Synchrotron radiation was employed in the case of **5f** due to its very small crystal size. For illustrative purposes, in this section, we discuss only the crystalline array of compounds **5a** and **5f** in detail. The relevant crystallographic data of all compounds and a detailed description of the crystal packing of compounds **5b** to **5e** are provided in Sections S3 and S5 in the Supporting Information.

**Figure 2:**
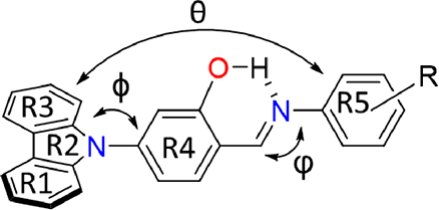
General structure of the *N*-salicylidene anilines in this work. Carbazole rings were labeled as R1, R2, and R3. The inner and outer phenyl rings were designated as R4 and R5, respectively. The ϕ angle is defined between the plane of carbazole ring R2 and the plane of ring R4, the φ angle lies between the planes of rings R4 and R5, and the θ is the angle between the planes of rings R2 and R5.

To provide a systematic description, we established some parameters that include the angle between the planes formed by the carbazole and central phenyl ring (ϕ), the angle between the planes of the central and outer phenyl rings (φ), the angle formed by the carbazole and outer phenylene (θ), and geometric parameters of the internal OH···N bond (Figure 2). Detailed information about these parameters can be found in Table 1.

**Table 1:** Conformational Parameters for the Asymmetric Units of Each Crystalline Structure^a^

**compound**	φ/°	**ϕ/°**	**θ/°**	**C–O/Å**	**∠OH···N/°**	**O–H/Å**	H···N**/Å**	O···N**/Å**
**5a**	57.91	14.97	72.72	1.352	152.7	0.98	1.67	2.571
**5b**	55.25	12.34	42.94	1.351	153.7	0.99	1.67	2.600
**5c**	55.26	12.11	43.38	1.351	150.0	0.98	1.73	2.622
**5d(i)**	56.06	25.07	81.10	1.349	153.7	0.95	1.73	2.621
**5d(ii)**	49.58	31.30	80.62	1.353	154.0	0.95	1.67	2.563
**5e(i)**	57.03	21.06	78.0	1.355	153.1	1.01	1.68	2.622
**5e(ii)**	49.55	34.31	83.46	1.352	150.8	1.03	1.68	2.631
**5f**	43.86	43.74	1.98	1.343	151.0	1.02	1.69	2.635

a Labels i and ii were added where two molecules were resolved per asymmetric unit.

**Figure 3:**
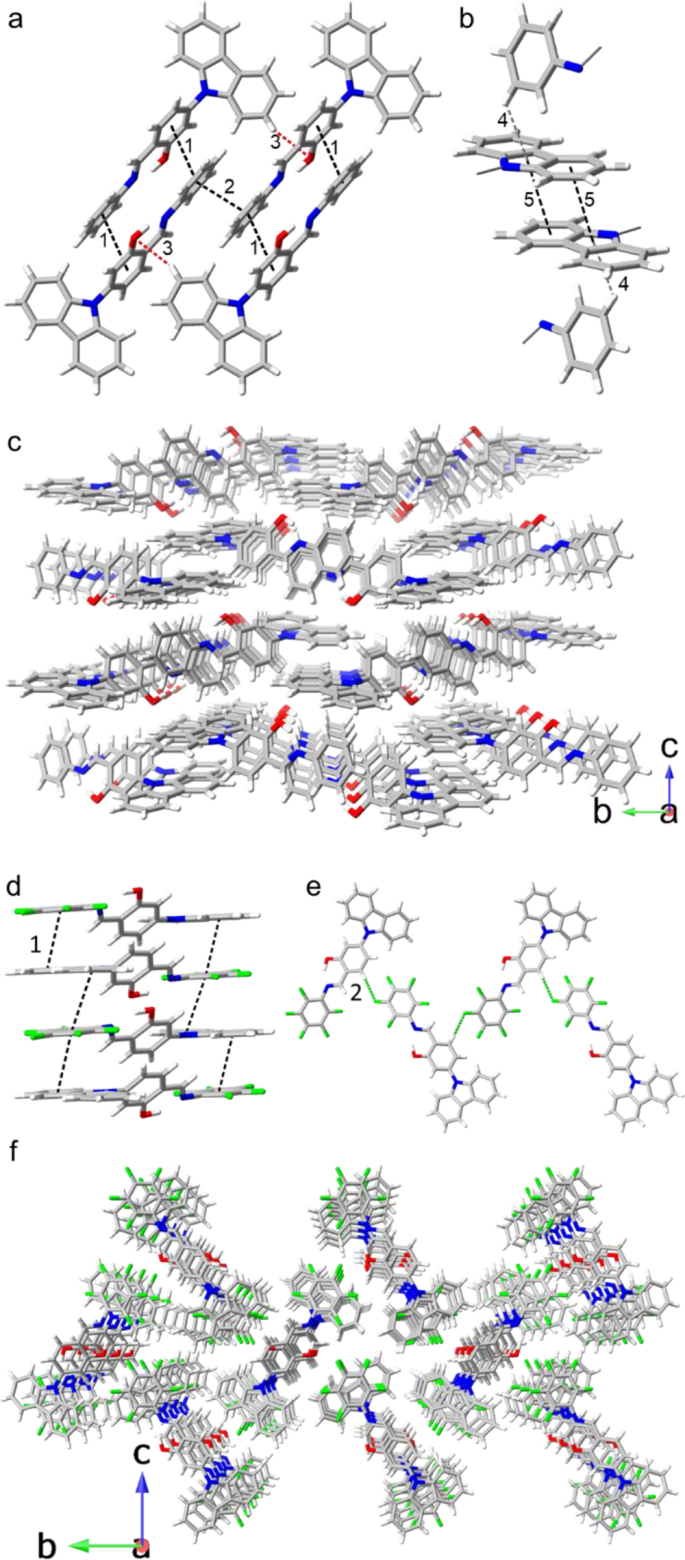
(a) π···π stacking in **5a** formed by phenylene rings through the [100] direction. (b) π···π and CH···π interactions propagate the crystal array through [001] and [010]. (c) Crystal packing of **5a** is viewed along the *a*-axis. (d) Head-to-tail π···π stacking arrangement of **5f**. (e) C–H···F interactions, which form a continuous chain in the crystal packing of **5g**. (f) Packing of **5f** viewed through the *a*-axis.

The structure of compound **5a** was solved in the *P*2_1_/c space group, with one molecule per asymmetric unit. This structure exhibited the smallest O···H distance for intramolecular hydrogen bonds. The crystal lattice of **5a** propagated through the [100] direction via a combination of π···π stacking and CH···O interactions (Figure 3a). Additionally, the array was further propagated through the [001] and [010] directions by π···π and CH···π interactions (Figure 3b). By comparison, compound **5f**, which was also solved in the *P*2_1_/c space group although is not isostructural to **5a** (Figure S6), exhibited the most considerable O–H distance in the intramolecular O–H···N hydrogen bonds. Furthermore, the θ angle shows a quasi-coplanar conformation facilitating the arene-perfluoroarene stacking in a head-to-tail fashion (Figure 3d) through the [001] direction, and a CH···F interaction propagates the lattice through the [010] direction (Figure 3e). These interactions are responsible for the crystal packing of the structure (Figure 3f, Table S2).

### Steady-State Fluorescence Studies in Solution and the Crystalline Solid State

Generally, Schiff base derivatives show a low emission intensity in solution and moderate emission in the solid state, and they are good bidentate groups that can chelate different metal ions. This aspect makes them excellent candidates to be used as turn-on/off sensors of Zn(II),^28,29^ Cu(II),^30^ Al(III),^31^ drug delivery,^32^ or for developing new AIEgens.^33^ In addition, there are a few *N*-salicylidene anilines in the literature with good emission in the solid state. For instance, Tong et al. reported a Schiff base derivative with a QY= 24.3%,^34^ and Wang et al. described a compound with a QY = 91.7%, both in the solid state.^35^

**Figure 4:**
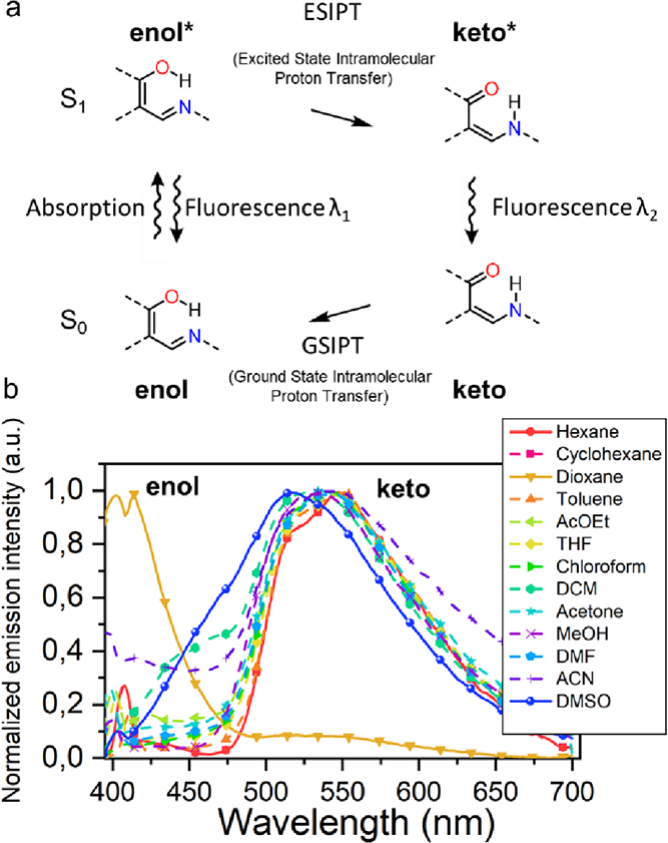
(a) Four-level photochemical cycle diagram for the ESIPT process. (b) Emission spectra of **5a** in 13 different solvents with different polarities.

It has been described that the intermolecular interactions between the solvent and the Schiff bases can modify the hydrogen–oxygen distance in the ground state.^36^ In favorable cases, UV–vis light irradiation is energetic enough to promote an excited state intramolecular proton transfer (ESIPT).^37^ Once this phenomenon occurs in photochromic *N*-salicylidene anilines, it gives rise to the photoinduced *keto* tautomer thanks to the ESIPT process as as a relaxation pathway in most cases followed by fluorescence at red-shifted wavelengths (Figure 4a).^38−41^ To determine if ESIPT fluorescence occurs and whether the formation of the *keto* form in compounds **5a**–**5f** follows an ESIPT fluorescence relaxation pathway, we evaluated the emission behavior by collecting the steady-state fluorescence (PL) response in 13 solvents with different polarities, which could stabilize either the *enol* or *keto* excited form. These solvents ranged from the apolar solvent (hexane, ε = 1.88) to the most polar solvent (DMSO, ε = 46.7), using the dielectric constant, ε, as a comparison parameter. It was observed that *enol* emission is favored in dioxane, while in other solvents, the *keto* form emission is present, as exemplified in Figure 4b for compound **5a**. The behavior was observed in all compounds, with a more noticeable contribution of the *keto* form in the compounds with a higher number of fluorine atoms (**5a** < **5b** < **5e** < **5f**) attributable to the increasingly lower basicity of the nitrogen atom. Their complete emission spectra can be found in Section S5a.

**Figure 5:**
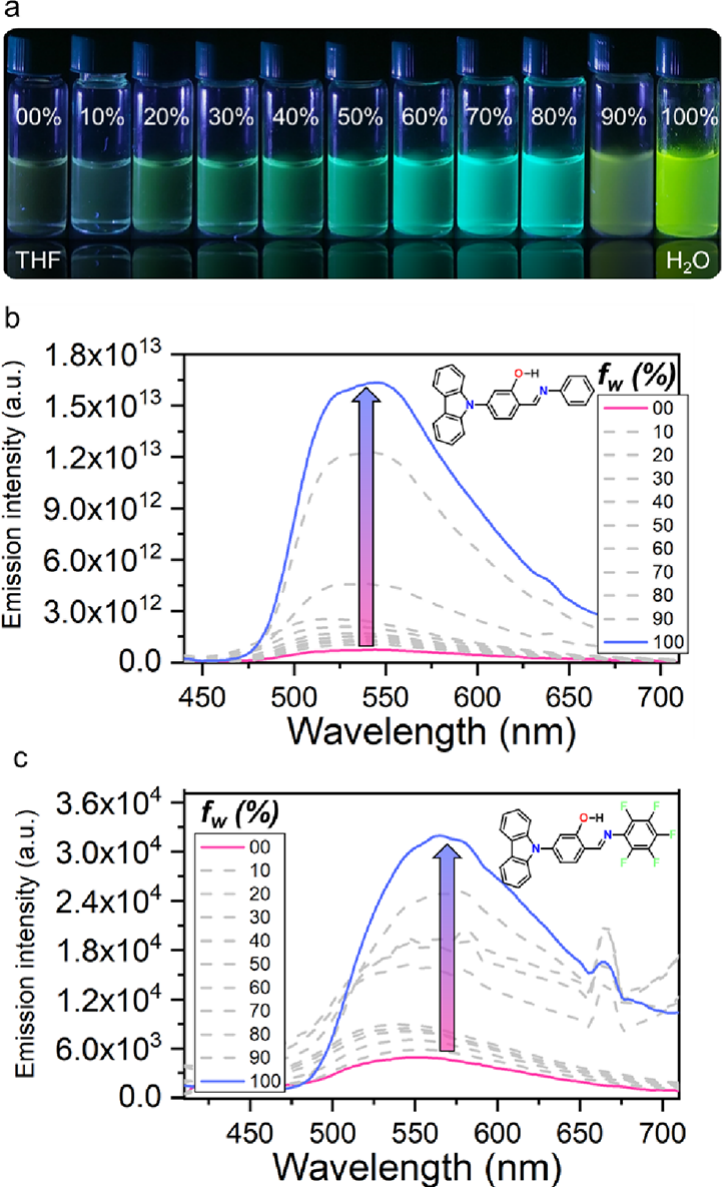
(a) Solution fluorescence response for **5a** in different THF:H_2_O mixtures. Aggregation-induced emission enhancement behavior comparison of compounds in different THF:H_2_O mixtures: (b) **5a** and (c) **5f**.

Despite showing emission in the explored solvents, the PL intensity of all compounds could be better in solution, which motivated us to explore their AIEE properties. We carried out the aggregation experiments by measuring the photoluminescence under different mixtures of THF:H_2_O (*f*_w_ = 0–100%) as a well-documented strategy to promote the formation of aggregates reducing the solubility of the studied molecule.^42^ Our studies show a subtle increase in the emission intensity on going from *f*_w_ = 0 to 60%. However, upon reaching *f*_w_ = 70%, an abrupt increment in the emission intensity was observed, especially for compound **5a** (Section S5), indicating that at higher water fractions, the compounds create highly emissive aggregates. In fact, in compound **5a**, the emission increase was observable by the naked eye (Figure 5a), with fluorescence measurements revealing an increment of 9 orders of magnitude (from 1.4 × 10^4^ to 1.7 × 10^13^ a.u.), as compared to that carried out in THF (Figure 5b). All of the compounds show AIEE behavior; nevertheless, in compound **5f**, the increase in fluorescence was only moderate (Figure 5c). Detailed information on AIEE behavior for compounds **5b**–**5e** can be found in Section S5b.

**Figure 6:**
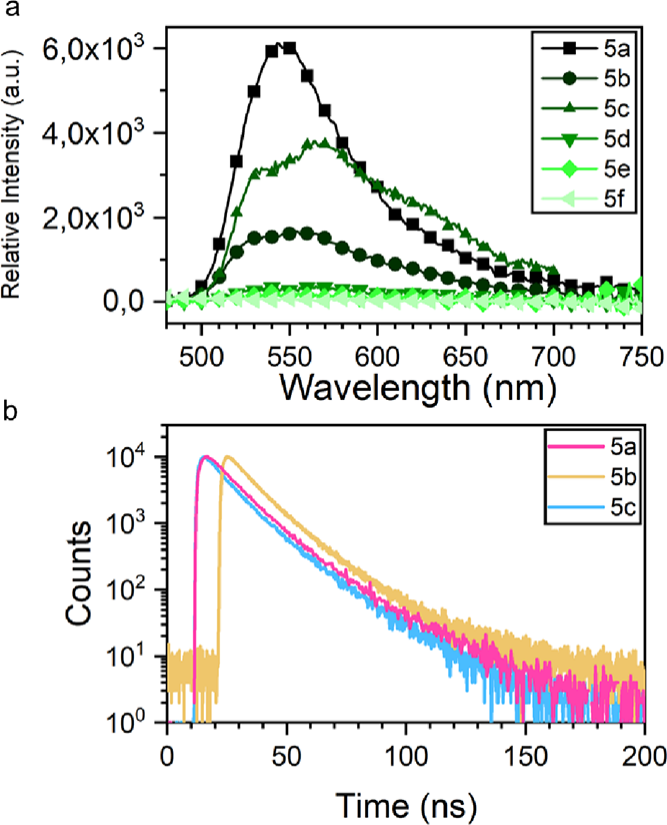
(a) Crystalline solid-state fluorescence spectrum of compounds **5a** to **5f**. (b) Time-resolved decay profiles for **5a** to **5c**.

Subsequently, we further investigated their fluorescence behavior in the crystalline solid state. We measured the crystalline solid-state fluorescence spectra, the fluorescence quantum yield (QY), and their fluorescence lifetimes (τ) (Figure 6). Fluorescence lifetime (FLT) measurements were performed on imines using the time-correlated single photon counting technique. The complete UV–vis and fluorescence spectroscopy data is available in Section S5. Table 2 summarizes the average FLT () for imines in solid state where τ_1_ and τ_2_ are referred to fast and slow constants, while *A*_1_ and *A*_2_ are the amplitude constants. Imines show τ values at the nanosecond time scale; fluorophores present multiexponential decays commonly observed for organic fluorophores in the solid state and are determined by intermolecular interactions and dynamics of excited states.^43^ The experimental photophysical data are summarized in Table 2. Compound **5a**, which does not possess fluorine atoms, exhibited the highest quantum yield (near 100%), and its fluorescence lifetime was adequately adjusted with a biexponential decay with τ_1_ and τ_2_ values of 11.57 and 26.06 ns, respectively. For compound **5b**, with a quantum yield of 30%, the lifetime was adjusted with τ_1_ = 9.50 ns and τ_2_ = 19.35 ns, whereas for compound **5c**, with a quantum yield of 24%, the lifetimes were τ_1_ = 10.35 ns and τ_2_ = 22.46 ns. In contrast, it was impossible to determine their fluorescence lifetimes for compounds **5e** and **5f** that present photochromism phenomena due to our instrument’s meager quantum yields and low resolution.

**Table 2:** Photophysical Properties of the Synthesized Compounds, Including Absorption and Emission Maxima, Quantum Yield, Fluorescence Lifetimes, Energy Difference of Frontier Molecular Orbitals, and Oscillator Strength

compound	absorption λ_max_ (nm)	emission λ_max_ (nm)	fluorescence QY (%)	τ_1_ (ns)	*A*_1_	τ_2_ (ns)	*A*_2_	τ_avg_ (ns)	ΔMO energy (eV)	oscillator strength
**5a**	362	541	99	11.57	85.58	26.06	14.42	13.65	5.99	0.829
**5b**	361	538	30	9.50	59.42	19.35	40.58	13.49	5.94	0.824
**5c**	364	568	24	10.35	72.69	22.46	27.31	13.65	5.84	0.817
**5d**	365	546	43	-a	-	-	-	-	5.86	0.830
**5e**	368	550	15	-	-	-	-	-	5.77	0.782
**5f**	373	551	<1	-	-	-	-	-	5.66	0.783

a - means not determined.

To understand this photophysical process better, we calculated the energy of the frontier molecular orbitals using Density Functional Theory (DFT) with the hybrid functional CAM-B3LYP and the 6-311++g(d,p) basis set. The increase in the number of fluorine atoms resulted in a slight reduction of the HOMO–LUMO energy gap, with a difference of 5.99 eV for compound **5a** to 5.66 eV for compound **5f** and oscillator strengths of 0.829 and 0.783 respectively (Table 2, Section S4). Results show that fluorescence QY undergoes a more drastic change. The QY reduction in **5f** concerning **5a** suggests additional relaxation pathways originated from replacing C–H for C–F. Such substitution implies an increased spin–orbit coupling,^44^ and electron-withdrawing effects of the C–F bonds, could stabilize nonradiative decay pathways, such as intersystem crossing, internal conversion, and vibrational relaxation.^45^ These factors collectively favor nonradiative transitions over fluorescence, leading to a decreased quantum yield. Another relaxation pathway that can occur is photoisomerization. To probe this idea, we decided to explore the photoisomerization behavior of these compounds through different strategies, as mentioned in the following section.

### Solid-State Photochromic Response

**Figure 7:**
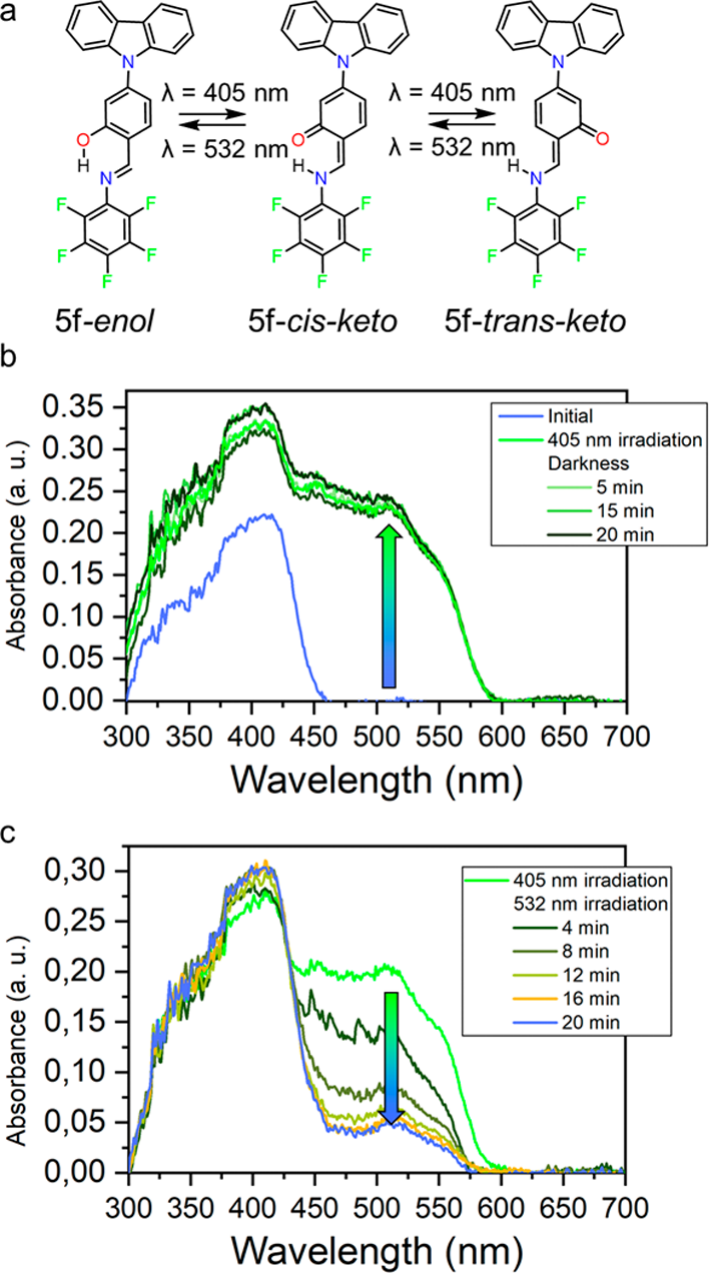
(a) Reversible *enol*–*keto* photochromic behavior of compound **5f**. (b) Formation and stability of **5f**-*trans-keto* form after irradiation with 405 nm and its stability in darkness. (c) Reversibility of the process after irradiation with 532 nm at different times.

*N*-Salicylidene anilines are studied by their switching performance.^13^ It is known that the aggregation phase influences the photochromic behavior of the *N*-salicylidene anilines, and it has been demonstrated that the photochromic behavior can take place in solution,^46^ thin films,^47^ after the introduction into a MOF matrix,^17^ or in crystalline but not in the amorphous solid state.^38^ Then, as a first approach to evaluate the photochromic behavior of compounds **5a**–**5f**, we used poly(methyl methacrylate) (PMMA) as a matrix to prepare the thin films. Despite numerous attempts, no discernible photochromic response was observed in the thin films. Furthermore, we tried melting the compounds between two quartz plates to obtain a vitreous solid and subsequently evaluated its photochromic response, but no photochromic response was observed. Finally, polycrystalline samples were placed between two quartz plates and irradiated with a 405 nm light source, revealing a photochromic response only for compounds **5e** (Figure S25) and **5f** (Figure 7). Therefore, the photochromic response for such compounds can only be facilitated by its crystalline array, supported by the fact that the φ angle is higher than 30° (Table 1).

We focused on sample **5f** for further evaluation and subjected it to light irradiation. Before irradiation, the samples exhibited an absorption maximum at 416 nm, which was attributed to the absorption of the *enol* form (Figure 7b). Upon irradiation with a 405 nm laser diode, a secondary band emerged at 516 nm, suggesting the presence of the *trans-keto* form. Remarkably, this form proved to be stable in the dark for at least 20 min and exhibited reversibility upon exposure to a 532 nm laser (Figure 7c).

Considering the X-ray diffraction data described above, it was possible to assess the potential photochromicity of the *N*-salicylidene anilines in their crystalline solid state. It has been reported that as the torsional angle φ deviates from coplanarity, the propensity for photochromism increases.^22,48,49^ As the data compiled in Table 1 show, the structures **5e** and **5f** exhibit the most pronounced φ angles, with 34.3 and 43.7°, respectively. In addition, for **5f**, there are no CH-acceptor adjacent interactions. Therefore, it is reasonable to expect that the photoisomerization process in **5f** could occur in the excited state.^50^

**Figure 8:**
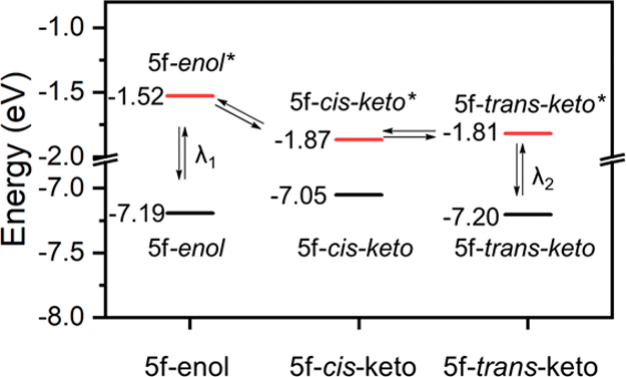
Energy levels for the calculated **5f**-*enol*, **5f**-*keto-cis*, and **5f**-*keto-trans*.

To get a more detailed understanding of the photoisomerization process, we calculated the frontier molecular orbital energies for the **5f**-*enol*, **5f**-*cis*-*keto*, and **5f**-*trans*-*keto* (Figure 8), implied in the photoisomerization process as illustrated in Figure 7a. Furthermore, we also calculated the UV–vis spectra through TD-DFT for such forms to investigate if the observed photoproduct of **5f** was the *keto-trans* species (Figure S10). Our computations indicate that the calculated absorbance for the **5f**-*keto-cis* is different from the **5f**-*keto*-*trans*, with a π–π* additional shoulder that agrees well with the solid-state absorption experimental data (Figure 7b). The stability of the **5f**-*keto-trans* form in darkness could be attributed to the high energy barrier in the ground state to reverse from the **5f**-*keto-trans* form to **5f**-*keto-cis*, with a calculated value of 43.4 kcal·mol^–1^ (Figure S11).

## Conclusions

Five carbazole *N*-salicylidene anilines **5a**–**5f** were synthesized using a two-step methodology involving sequential Ullmann coupling and imine condensation. The fluorescence experiments in solution revealed a gradual decrease in the emission intensity with the inclusion of fluorine atoms, going quantum yields near unity for compound **5a**, to an almost quenched fluorescence in compound **5f**, suggesting that the fluorine substitution opens an alternative relaxation pathway. Additionally, all the compounds show excited state intramolecular proton transfer (ESIPT) fluorescence. Last, the aggregation-induced emission enhancement (AIEE) properties of all compounds were explored, revealing that the nonfluorinated compound shows a stronger increase in emission intensity, with a diminished fluorescence response observed with the introduction of fluorine atoms.

The inclusion of fluorine atoms is advantageous because it drives the arene-perfluoroarene intermolecular interactions, thus influencing the crystallization and supramolecular array of the compounds reported here. In addition, only trifluorinated **5e** and pentafluorinated compound **5f** exhibited a photochromic response in the crystalline solid state when irradiated with a 405 nm laser diode, attributed to the *enol-cis* to *keto-trans* photoisomerization. The **5f**-*keto-trans* form can be reversed to the **5f**-*enol* form by using a 532 nm laser source. Our X-ray and computational data indicate that this response is possible due to the high torsional angle between the central phenylene ring and the aniline.

Our findings provide a detailed examination of the effect of fluorine substitution in the emission response of this series, shedding light on compounds with potential applications such as ESIPT, AIEEgens, or solid-state photoswitches, leading the way to the design and optimization of crystalline materials with tailored photophysical properties.

## Supporting Information Available

Synthesis conditions, ^1^H NMR, ^13^C NMR, IR spectra data and HRMS of each compound, X-ray collection details, DFT calculations and UV–vis and Fluorescence data in solution and the solid state (PDF)

## Accession Codes

CCDC 2347348–2347353 contain the supplementary crystallographic data for this paper. These data can be obtained free of charge via www.ccdc.cam.ac.uk/data_request/cif, or by emailing data_request@ccdc.cam.ac.uk, or by contacting The Cambridge Crystallographic Data Centre, 12 Union Road, Cambridge CB2 1EZ, UK; fax: + 44 1223 336033.
