# Supplementary material for: Aggregation-Induced Emission Enhancement and Solid-State Photoswitching of Crystalline Carbazole N-Salicylidene Anilines
Source: ACS Omega. 2024 Aug 23;9(36):38015–22. doi: 10.1021/acsomega.4c04764 (PMC11391533; doi:10.1021/acsomega.4c04764)
Supplement: Supplementary file 1 — ao4c04764_si_001.pdf [file ao4c04764_si_001.pdf]

Supporting Information

Aggregation Induced Emission Enhancement (AIEE) and Solid-State Photoswitching of Crystalline Carbazole *N*-salicylideneanilines

Dazaet Galicia-Badillo,[1] José L. Belmonte-Vázquez,[2] Mario Rodríguez,[3] Braulio Rodríguez-Molina[1] and Ma. Carmen García-González[1]**

1. Instituto de Química (IQ), Universidad Nacional Autónoma de México (UNAM), Circuito Exterior s/n, Ciudad Universitaria, Coyoacán, 04510, Ciudad de México, México.

2. Departamento de Química Orgánica, Facultad de Química (FQ), Universidad Nacional Autónoma de México (UNAM), Ciudad Universitaria, Ciudad de México 04510, México.

3. Research Group of Optical Properties of Materials (GPOM), Centro de Investigaciones en Óptica, CIO, A.P. 1-948, León, Guanajuato 37000, México

brodriguez@iquimica.unam.mx
carmen.garcia@iquimica.unam.mx

Contents

S1. Material and methods 3

S2. General procedure for the synthesis of *N*-Salicylideneanilines 3

S3. X-Ray diffraction studies 12

a. Structures 5b and 5c 14

b. Structures 5d and 5e 16

S4. Density Functional Theory Studies 18

S5. Steady-State Photophisycal measurement studies 20

a. Solution Steady-State absorption and emission in different solvents....21

b. Solution Steady-State absorption and emission in different H_2O:THF mixtures 25

S1. Material and methods

All reagents were purchased from Sigma-Aldrich and used as received. Flash column chromatography was performed using Aldrich silica gel 230-400 mesh, hexane and ethyl acetate as eluents. Reactions were monitored by TLC on silica gel plates 60 F254 (Merck) and the spots were detected by UV-absorption. ^{1}H and ^{13}C NMR spectra were recorded at ambient temperature using Bruker Fourier 300, and Jeol Eclipse 300 spectrometers, chemical shifts (δ) are reported in ppm relative to the solvent signal, with an internal TMS standard, NMR coupling constants are reported in hertz (Hz). The FT-IR spectral data were recorded with Bruker ATR in the 450-4000 cm-1 range. High-resolution mass spectra were recorded on a JEOL AccuTOF JMS-T100LC mass spectrometer by Direct Analysis in Real Time (DART). Melting points were determined using Fisher Johns melting point apparatus (uncorrected). For solid-state fluorescent measurements, an FS5 Edinburgh Instruments spectrofluorometer was employed. For photoisomerization experiments diode lasers 405 nm (57 mW), and 532 nm (mW) were employed.

S2. General procedure for the synthesis of *N*-Salicylideneanilines

Equimolar quantities of 4-(9*H*-carbazol-9-yl)-2-hydroxybenzaldehyde and the appropriate amine were added into a round-bottom flask using methanol (10 mL), the reaction was stirred under reflux for 24 h, the solid precipitated was collected by filtration and washed with hexanes.

5-(9*H*-carbazol-9-yl)-2-((phenylimino)methyl)phenol (5a)

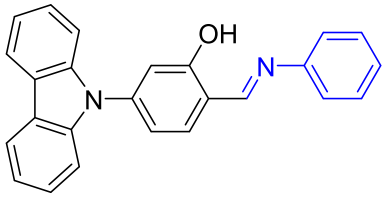

The title compound was prepared according to general procedure from **3** (0.030g, 0.104 mmol) and aniline (0.010g, 0.107 mmol) to give **5a** (0.027g, 73 % yield) as a solid yellow, m.p. 109 - 110 °C.

IRν_{max}: 3065, 3025, 1603, 1537, 1509, 1443, 1199 cm^{-1}.

^{1}H NMR (300 MHz, $CDCl_3$) δ: 13.71 (s, 1H), 8.72 (s, 1H), 8.13 (dd, 2H, *J*=0.6, 7.8 Hz), 7.61-7.57 (m, 3H), 7.49-7.41 (m, 4H), 7.35-7.28 (m, 6H), 7.19 (dd, 1H, *J*=2.1, 8.1 Hz).

^{13}C NMR (75 MHz, $CDCl_3$) δ: 162.8, 161.9, 148.4, 142.2, 140.4, 133.7, 129.7, 127.3, 126.3, 124.0, 121.4, 120.6, 120.5, 118.2, 117.3, 115.1, 110.4.

HRMS (DART+) calculated for $C_{25}H_{19}N_2O$ $[M+H]^+$ 363.1497; found 363.1499.

5-(9*H*-carbazol-9-yl)-2-(((4-fluorophenyl)imino)methyl)phenol (5b)

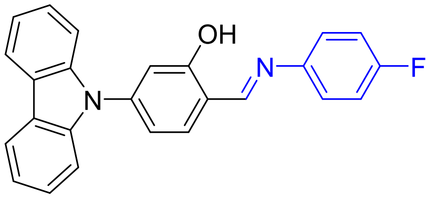

The title compound was prepared according to general procedure from **3** (0.150g, 0.522 mmol) and 4-fluoroaniline (0.058 g, 0.522 mmol) to give **5b** (0.129 g, 65 % yield) as a solid yellow, m.p. 196-197 °C.

IRν_{max}: 3227, 3058, 2930, 2853, 1657, 1484, 1250, 469 cm^{-1}.

^{1}H NMR (300 MHz, $CDCl_3$) δ: 7.46 (d, 2H, *J*= 9.0 Hz), 7.38-7.29 (m, 6H), 7.13-7.01 (m 5H), 6.72 (d, 1H, *J*=15 Hz), 6.31 (d, 1H, *J*=6 Hz), 5.50 (s, 1H), 3.88-3.79 (m,1H), 2.02-1.96 (m, 2H), 1.73-1.57 (m, 3H), 1.39-1.18 (m, 4H), 0.89-0.82 (m, 1H).

^{13}C NMR (75 MHz, $CDCl_3$) δ: 168.4, 167.7, 139.4, 137.7, 137.4, 136.7, 135.6, 132.3, 131.1, 130.0, 128.6, 128.5, 128.3, 126.7,126.6, 121.9, 119.2, 93.8, 63.9, 48.6, 32.6, 25.3, 24.6. **HRMS** (DART+) calculated for $C_{25}H_{18}FN_2O$ $[M+H]^+$ 381.1403; found 381.1401.

5-(9*H*-carbazol-9-yl)-2-(((3,4-difluorophenyl)imino)methyl)phenol (5c)

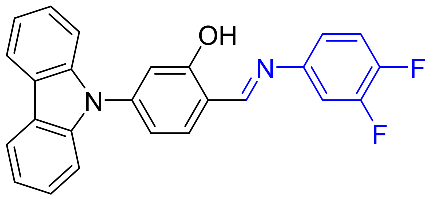

The title compound was prepared according to general procedure from **3** (0.150g, 0.522 mmol) and 3,4-difluoroaniline (0.067 g, 0.522 mmol) to give **5c** (0.142 g, 68 % yield) as a solid yellow, m.p. 199-200 °C.

IRν_{max}: 3052, 2922, 1620, 1601, 1514, 1450, 1197 cm^{-1}.

^{1}H NMR (300 MHz, $CDCl_3$) δ: 7.46 (d, 2H, *J*= 9.0 Hz), 7.38-7.29 (m, 6H), 7.13-7.01 (m 5H), 6.72 (d, 1H, *J*=15 Hz), 6.31 (d, 1H, *J*=6 Hz), 5.50 (s, 1H), 3.88-3.79 (m,1H), 2.02-1.96 (m, 2H), 1.73-1.57 (m, 3H), 1.39-1.18 (m, 4H), 0.89-0.82 (m, 1H).

^{13}C NMR (75 MHz, $CDCl_3$) δ: 168.4, 167.7, 139.4, 137.7, 137.4, 136.7, 135.6, 132.3, 131.1, 130.0, 128.6, 128.5, 128.3, 126.7,126.6, 121.9, 119.2, 93.8, 63.9, 48.6, 32.6, 25.3, 24.6. **HRMS** (DART+) calculated for $C_{25}H_{17}F_2N_2O$ $[M+H]^+$ 399.1309; found 399.1313.

5-(9*H*-carbazol-9-yl)-2-(((3-chloro-4-fluorophenyl)imino)methyl)phenol (5d)

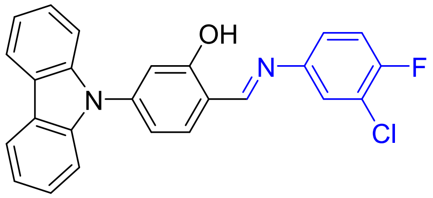

The title compound was prepared according to general procedure from **3** (0.030g, 0.104 mmol) and 3-chloro-4-fluoroaniline (0.015 g, 0.104 mmol) to give **5d** (0.034 g, 79 % yield) as a solid yellow, m.p. 185-186 °C.

IRν_{max}: 3227, 3058, 2930, 2853, 1657, 1484, 1250, 469 cm^{-1}.

^{1}H NMR (300 MHz, $CDCl_3$) δ: 7.46 (d, 2H, *J*= 9.0 Hz), 7.38-7.29 (m, 6H), 7.13-7.01 (m 5H), 6.72 (d, 1H, *J*=15 Hz), 6.31 (d, 1H, *J*=6 Hz), 5.50 (s, 1H), 3.88-3.79 (m,1H), 2.02-1.96 (m, 2H), 1.73-1.57 (m, 3H), 1.39-1.18 (m, 4H), 0.89-0.82 (m, 1H).

^{13}C NMR (75 MHz, $CDCl_3$) δ: 168.4, 167.7, 139.4, 137.7, 137.4, 136.7, 135.6, 132.3, 131.1, 130.0, 128.6, 128.5, 128.3, 126.7,126.6, 121.9, 119.2, 93.8, 63.9, 48.6, 32.6, 25.3, 24.6. **HRMS** (DART+) calculated for $C_{25}H_{17}ClFN_2O$ $[M+H]^+$ 415.1013; found 415.1014.

5-(9*H*-carbazol-9-yl)-2-(((3,4,5-trifluorophenyl)imino)methyl)phenol (5e)

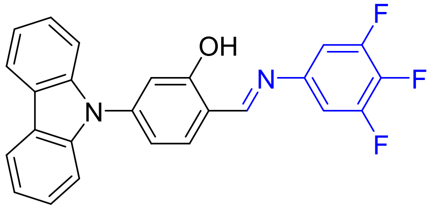

The title compound was prepared according to general procedure from **3** (0.15 g, 0.522 mmol) and 3,4,5-trifluoroaniline (0.077g, 0.523 mmol) to give **5e** (0.153 g, 71 % yield) as a solid yellow, m.p. 207 - 209 °C.

IRν_{max}: 3227, 3058, 2930, 2853, 1657, 1484, 1250, 469 cm^{-1}.

^{1}H NMR (300 MHz, $CDCl_3$) δ: 7.46 (d, 2H, *J*= 9.0 Hz), 7.38-7.29 (m, 6H), 7.13-7.01 (m 5H), 6.72 (d, 1H, *J*=15 Hz), 6.31 (d, 1H, *J*=6 Hz), 5.50 (s, 1H), 3.88-3.79 (m,1H), 2.02-1.96 (m, 2H), 1.73-1.57 (m, 3H), 1.39-1.18 (m, 4H), 0.89-0.82 (m, 1H).

^{13}C NMR (75 MHz, $CDCl_3$) δ: 168.4, 167.7, 139.4, 137.7, 137.4, 136.7, 135.6, 132.3, 131.1, 130.0, 128.6, 128.5, 128.3, 126.7,126.6, 121.9, 119.2, 93.8, 63.9, 48.6, 32.6, 25.3, 24.6. **HRMS** (DART+) calculated for $C_{25}H_{16}F_3N_2O$ $[M+H]^+$ 417.1215; found 417.1223.

5-(9*H*-carbazol-9-yl)-2-(((perfluorophenyl)imino)methyl)phenol (5f)

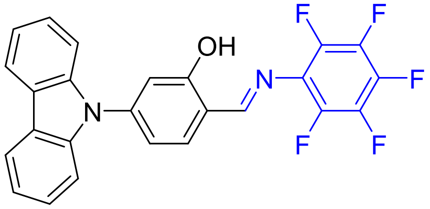

The title compound was prepared according to general procedure from **3** (0.057 g, 0.198 mmol) and 2,3,4,5,6-pentafluoroaniline (0.036 g, 0.198 mmol) to give **5f** (0.045 g, 51 % yield) as a solid yellow, m.p. 260-261 °C.

IRν_{max}: 2954, 2921, 1614, 1518, 1508, 1448, 1224 cm^{-1}.

^{1}H NMR (700 MHz, $CDCl_3$) δ: 12.56 (s, 1H), 8.86 (s, 1H), 8.07 (d, 2H, *J*= 7.7 Hz), 7.56 (d, 1H, *J*= 8.4 Hz), 7.53 (d, 2H, *J*= 7.7 Hz), 7.38 (ddd, 2H, *J*= 0.7, 1.4, 7.7 Hz), 7.27-7.25 (m, 3H), 7.20 (d, 1H, *J*= 2.1 Hz).

^{13}C NMR (175 MHz, $CDCl_3$) δ: 169.6, 163.1, 144.0, 140.1, 134.8, 126.4, 124.2, 120.9, 120.6, 117.6, 117.5, 115.2, 110.4.

HRMS (DART+) calculated for $C_{25}H_{14}F_5N_2O$ $[M+H]^+$ 453.1026; found 453.1013.

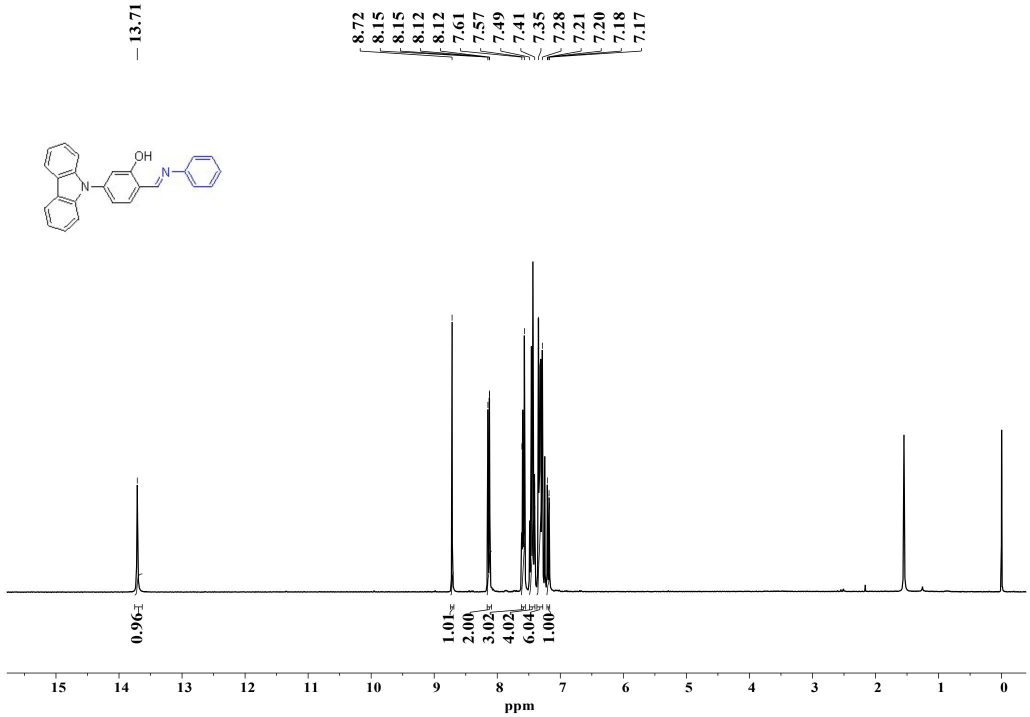

Figure S 1. ^{1}H NMR spectrum of **5a** (300 MHz, $CDCl_3$).

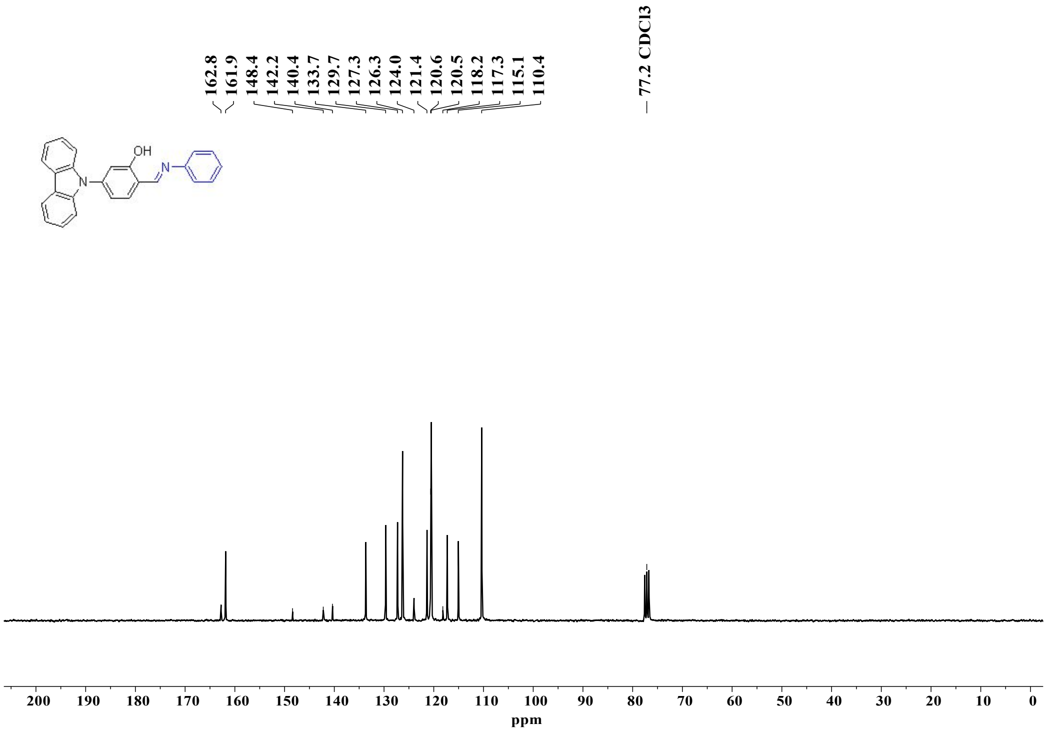

Figure S 2. ^{13}C NMR spectrum of **5a** (300 MHz, $CDCl_3$).

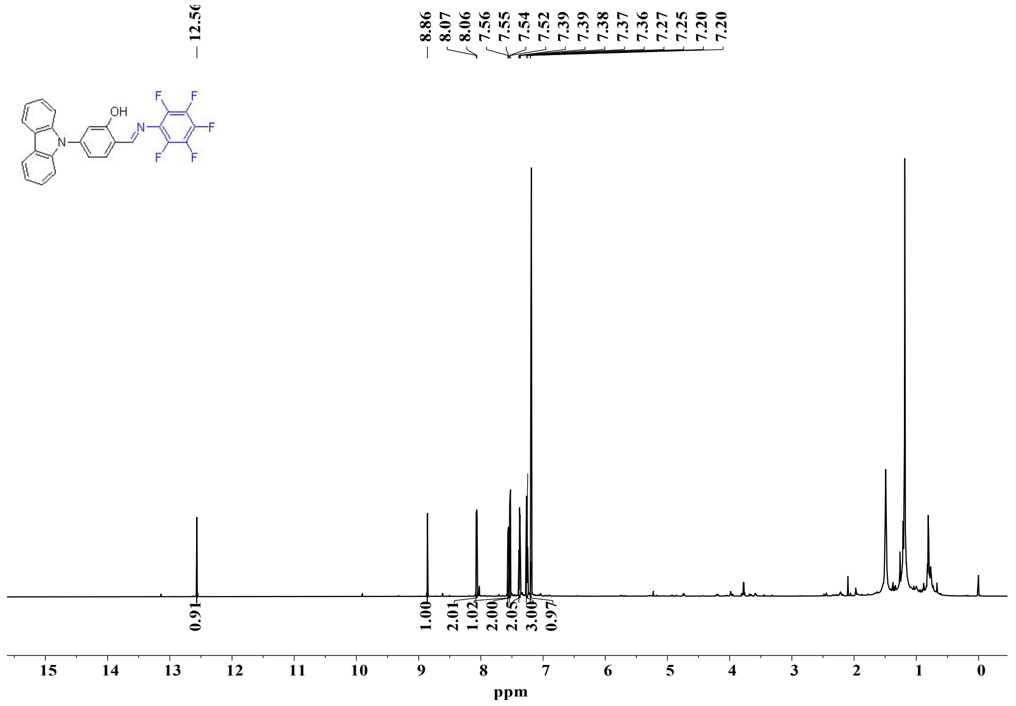

Figure S 3. ^{1}H NMR spectrum of **5f** (75 MHz, $CDCl_3$).

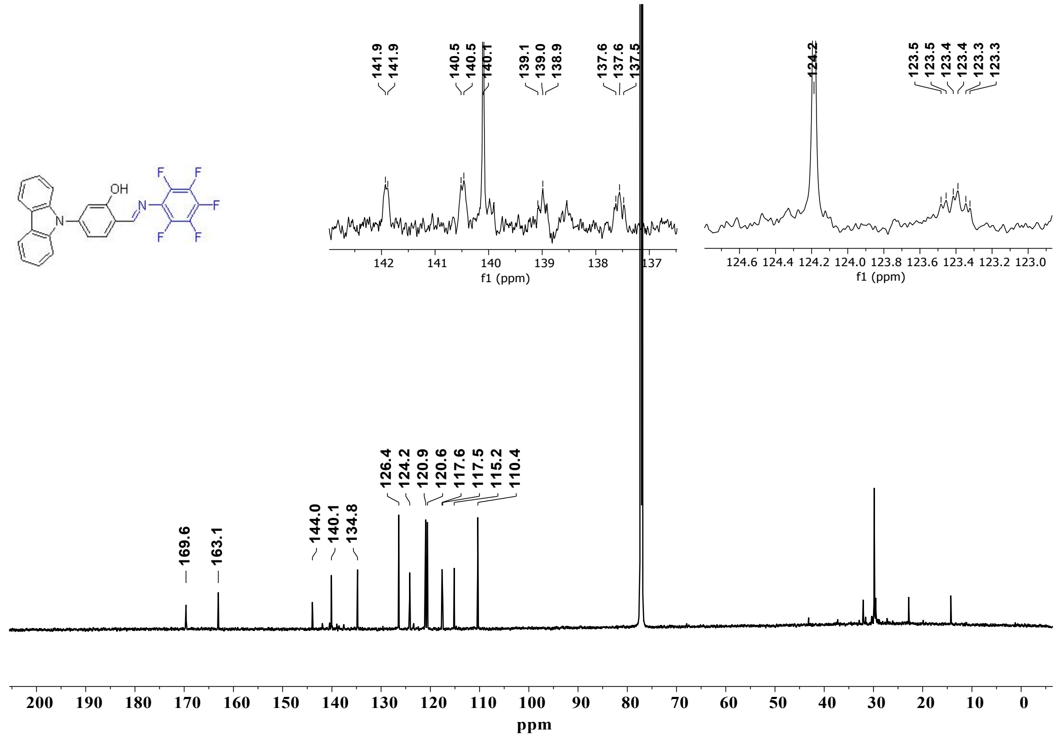

Figure S 4. ^{13}C NMR spectrum of **5f** (75 MHz, $CDCl_3$).

S3. X-Ray diffraction studies

Data collection was performed at 150 K and 298 K on a Bruker-D8-Venture diffractometers with Cu Kα-radiation, λ = 1.54178 Å. The structures were solved by direct methods and refined using SHELXL-2014. Non-hydrogen atoms were refined anisotropically.

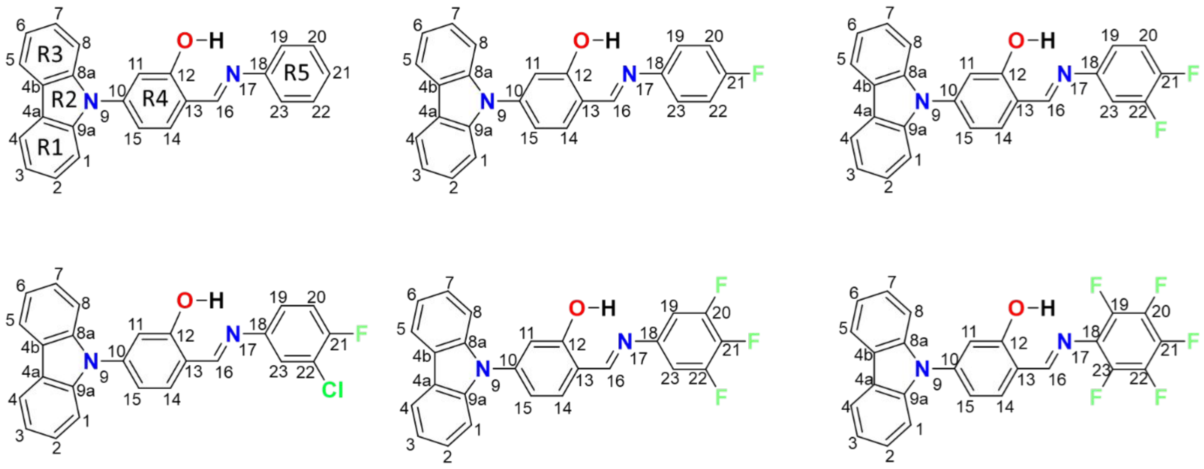

Figure S 5. Detailed system numbering for each structure used in this work. In structures with two molecules per asymmetric unit atoms were labeled with A or B as suffix.

Table S 1. Relevant crystallographic parameters for crystals **5a**-**5f**

**Schiff base**	**5a** 026RMB19	**5b** 341RMB18	**5c** 401RMB18	**5d** 343RMB18	**5e** 270RMB18	**5f** **3f(300)**
Formula	$C_{25}H_{18}N_2O$	$C_{25}H_{17}FN_2O$	$C_{25}H_{16}F_2N_2O$	$C_{25}H_{16}ClFN_2O$	$C_{25}H_{15}F_3N_2O$	$C_{25}H_{13}F_5N_2O$
MW/gmol^{-1}	362.41	380.40	398.40	414.85	416.39	452.37
T/K	150	298	150	298	298	298
Crystal system	Monoclinic	Monoclinic	Monoclinic	Monoclinic	Monoclinic	Monoclinic
Space group	$P\ 2_1/c$	$P\ 2_1/c$	$P\ 2_1/c$	$P\ 2_1/c$	$P\ 2_1/c$	$P\ 2_1/c$
a/Å	8.4867(3)	8.2808(6)	8.0451(3)	8.6590(19)	8.7209(8)	7.3860(2)
b/Å	16.6089(7)	29.2998(18)	31.0551(13)	35.176(8)	34.587(3)	16.9744(5)
c/Å	12.9692(6)	8.1098(5)	7.5264(3)	13.341(3)	13.3094(12)	15.6381(5)
α(°)	90	90	90	90	90	90
β(°)	98.164(1)	103.201(2)	98.914(1)	97.172(6)	97.864(3)	92.2270(10)
γ(°)	90	90	90	90	90	90
V/Å^3	1809.54(13)	1915.7(2)	1857.69(13)	4031.7(16)	3976.8(6)	1959.11(10)
Z	4	4	4	8	8	4
Z’	1	1	1	2	2	1
ρ/g cm^{-3}	1.330	1.319	1.424	1.367	1.391	1.534
μ/mm^{-1}	0.082	0.088	0.102	0.218	0.105	0.134
F(000)	760	792	824	1712	1712	920
Radiation/Å	MoKα 0.71073	MoKα 0.71073	MoKα 0.71073	MoKα 0.71073	MoKα 0.71073	0.7288 Å
Reflections Collected	31177	35474	26172	59072	81257	120345
Independent Reflections	5057 R(int) = 0.0974	5143 R(int) = 0.1714	5218 R(int) = 0.0963	8535 R(int) = 0.1128	9907 R(int) = 0.1062	4699 [R(int) =0.0465]
Data / restraints / parameters	5057 / 0 / 256	5143 / 0 / 265	5218 / 0 / 275	8535 / 437 / 648	9907 / 0 / 568	4699 / 0 / 302
Goodness of fit on F^2	1.011	1.019	1.015	1.001	0.998	1.132
Final R indices [I>2σ(I)]	R1 = 0.0572 wR2 = 0.0998	R1 = 0.0653 wR2 = 0.1145	R1 = 0.0577 wR2 = 0.0910	R1 = 0.0592 wR2 = 0.1125	R1 = 0.0580 wR2 = 0.1005	R1 = 0.0443, wR2 = 0.1215
R índices (all data)	R1 = 0.1411 wR2 = 0.1259	R1 = 0.1607 wR2 = 0.1481	R1 = 0.1532 wR2 = 0.1147	R1 = 0.1874 wR2 = 0.1503	R1 = 0.1721 wR2 = 0.1336	R1 = 0.0558, wR2 = 0.1286

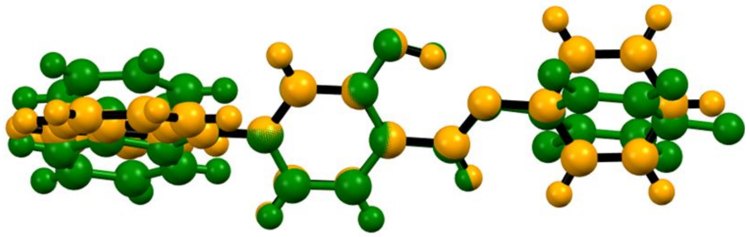

Figure S 6. Overlay of **5a** (orange) and **5f** (green) asymmetric unities.

a. Structures 5b and 5c

The crystal arrays of these structures display similarities in their structures. Both structures were resolved in a $P2_1/c$ space group with one molecule per asymmetric unit and have similar φ, ϕ, and θ angles. Specifically, the crystal structures propagate through the [101] direction due to the presence of CH···π, CH···O, and CH···F interactions (Figure S 7a,d). On the other hand, the crystal structures propagate through the [010] direction because of a ladder-type arrangement produced by carbazole moieties (Figure S 7b,e). Detailed geometric parameters of supramolecular interactions could be found in **Error! Reference source not found.**. Notably, despite the incorporation of an additional fluorine atom in the **5c** structure, similar interactions and geometries govern the crystal packing (Figure S 7c,f).

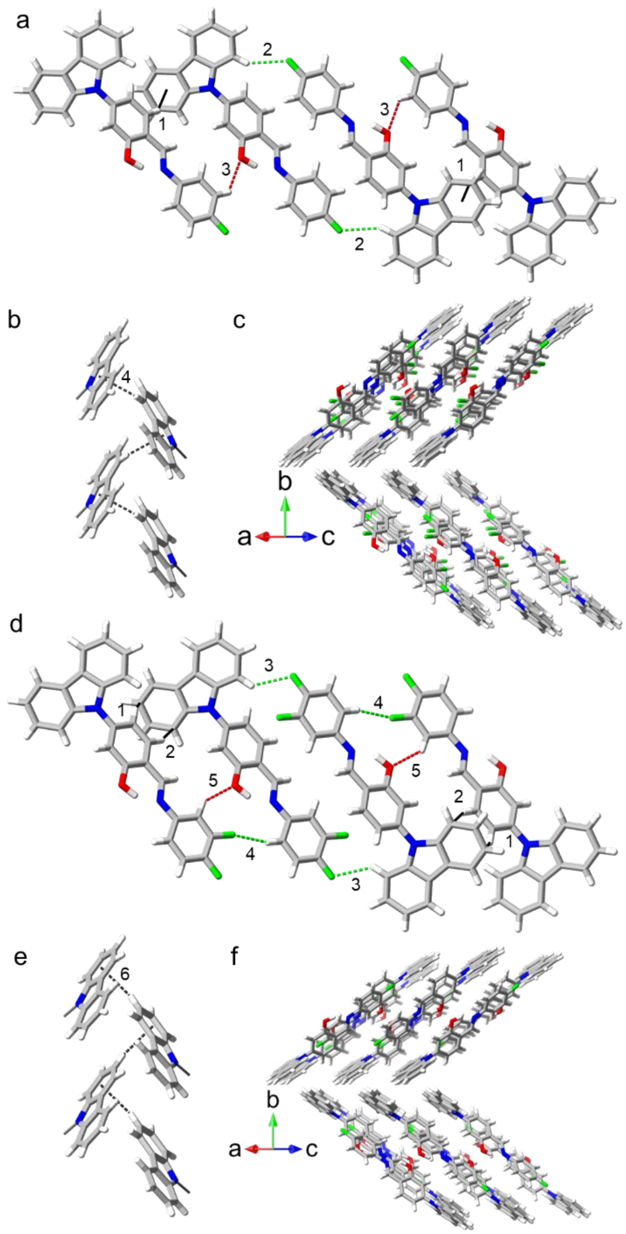

Figure S 7. Supramolecular motifs from **5b** and **5c**. a) Chains formed by CH···π, CH···O, CH···F hydrogen bonds of **5b**. b) CH···π chains with carbazole moieties **5b**. c) Packing of **5b** viewed through [101] direction. d) Chains formed by CH···π, CH···O, CH···F hydrogen of **5c**. e) CH···π chains with carbazole moieties **5c**. f) Packing of **5c** viewed through [101] direction.

b. Structures 5d and 5e

Structures **5d** and **5e** share similarities as they were both determined in the $P2_1/c$ space group with comparable cell parameters and φ, ϕ, and θ angles. Although **5d** has a disordered outer phenylene moiety with the chlorine atom in two flipped positions, the analysis was carried out using only the moiety with the major occupancy. Notably, the presence of two molecules per asymmetric unit in both structures leads to a significant increase in the number of different supramolecular interactions compared to previous structures. Each asymmetric unit forms a distinct layer through CH···O interactions (Figure S 8a,d), and these layers are joined by CH···π interactions from carbazole moieties (Figure S 8b,e), resulting in similar crystal packing for both structures (Figure S 8c,f).

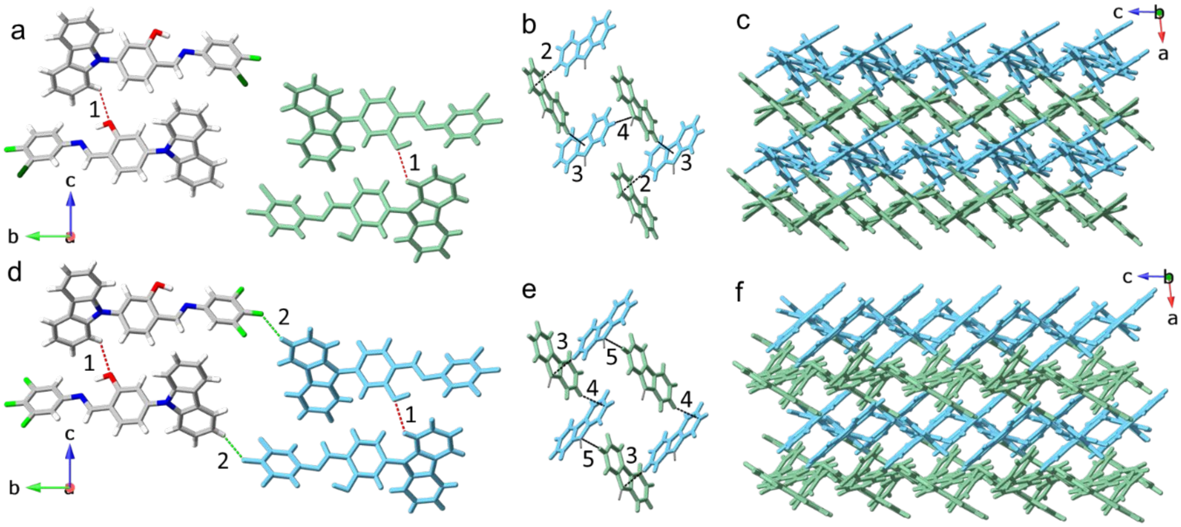

Figure S 8. Supramolecular motifs from **5d** and **5e**. a) Chains of CH···O bonds formed by one asymmetric molecule of **5d**. b) Carbazole CH···π chains formed by the two asymmetric molecules of **5d**. c) Packing of **5d** viewed through b axis. d) Chains of CH···O bonds formed by one asymmetric molecule of **5e**. e) Carbazole CH···π chains formed by the two asymmetric molecules of **5e**. f) Packing of **5e** viewed through b axis.

Table S 2. Bond distances and angles for supramolecular interactions D-X···A and π···π, of structures **5a-5h**.

Structure	#	Interaction	D-X (Å)	X···A (Å)	X···A (Å)	∠DXA (deg)	Centroide-Centroide (Å)
**5a**	1	π(R4$_{cen}$)···π (R5$_{cen}$)	-	-	-	-	3.831
	2	π(R5$_{cen}$)··· π(R5$_{cen}$)	-	-	-	-	3.548
	3	C(8)-H(8)···O	0.95	2.76	3.65	156.0	-
	4	C(20)-H(20)···π(R2$_{cen}$)	0.95	2.75	3.47	132.9	-
	5	π(R1$_{cen}$)···π(R2$_{cen}$)	-	-	-	-	3.671
**5b**	1	C(14)-H(14)···π(R3$_{cen}$)	0.93	2.83	3.60	141.6	-
	2	C(1)-H(1)···F(21)	0.93	2.64	3.47	129.7	-
	3	C(22)-H(22)···O	0.93	2.69	3.37	130.5	-
	4	C(5)-H(5)··· π(R2$_{cen}$)	0.93	2.88	3.61	136.8	-
**5c**	1	C(15)-H(15) ··· π(C3)	0.95	2.92	3.59	128.6	-
	2	C(14)-H(14) ··· π(C1)	0.95	2.90	3.69	140.9	-
	3	C(1)-H(1) ···F(21)	0.95	2.48	3.20	133.2	-
	4	C(19)-H(19)···F(22)	0.95	2.56	3.48	163.3	-
	5	C(23)-H(23)···O	0.95	2.60	3.38	139.3	-
	6	C(6)-H(6) ··· π(R3$_{cen}$)	0.95	2.75	3.62	152.2	-
**5d**	1	C(1*ii*)-H(1*ii*)···O*i*	0.93	2.64	3.27	126.3	-
	2	C(2*i*)-H(2*i*)···π(C1*ii*)	0.93	3.01	3.93	174.1	-
	3	C(7*ii*)-H(7*ii*)···π(R2*i*)	0.93	2.79	3.61	147.0	-
	4	C(7*i*)-H(7*i*)···π(N*ii*)	0.93	2.80	3.73	175.5	-
**5e**	1	C(1*i*)-H(1*i*)···O*i*	0.93	2.63	3.29	128.0	-
	2	C(3*i*)-H(3i)···F(21*i*)	0.93	2.63	3.33	132.6	-
	3	C(7*i*)-H(7i)···π(N*ii*)	0.93	2.95	3.86	166.6	-
	4	C(2*ii*)-H(2ii)··· π(C1*i*)	0.93	2.95	3.79	149.5	-
	5	C(7*ii*)-H(7ii)···π(N*i*)	0.93	2.78	3.71	172.6	-
**5f**	1	π(R3$_{cen}$)··· π(R5$_{cen}$)	-	-	-	-	3.67
	2	π(R2$_{cen}$)··· π(R2$_{cen}$)	-	-	-	-	3.98
	3	C(21)-F(21)···O	1.34	2.93	3.68	113.5	-

S4. Density Functional Theory Studies

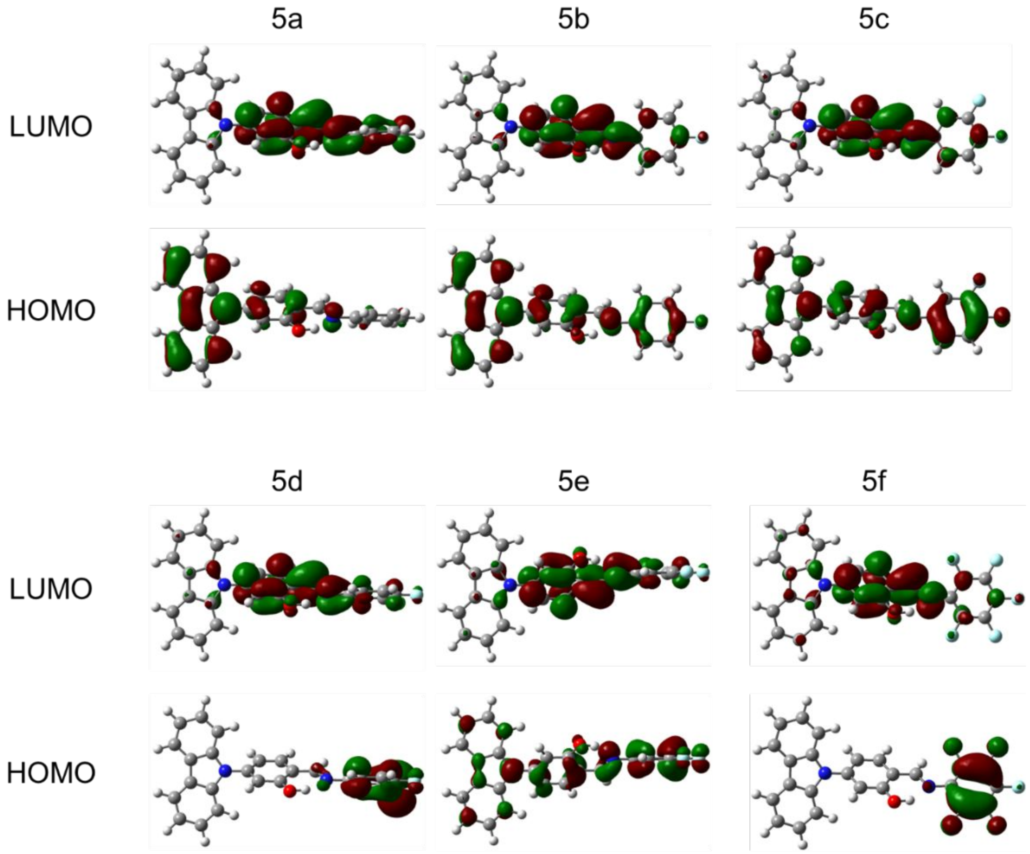

Figure S 9. Calculated Frontier Molecular Orbitals HOMO-LUMO for each molecule.

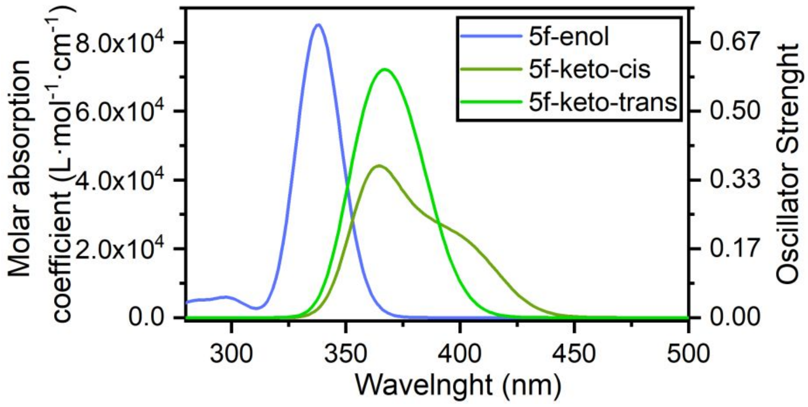

Figure S 10. Calculate UV-Vis spectra of **5f**-enol, **5f**-keto-cis and **5f**-keto-trans.

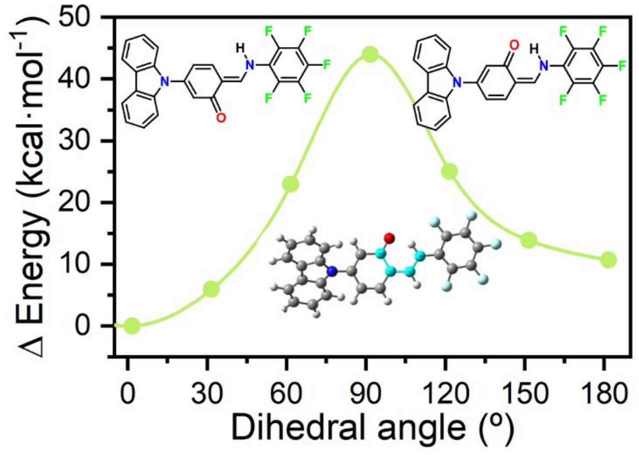

Figure S 11. Potential Energy Surface of **5f** as function of dihedral angle.

S5. Steady-State Photophisycal measurement studies

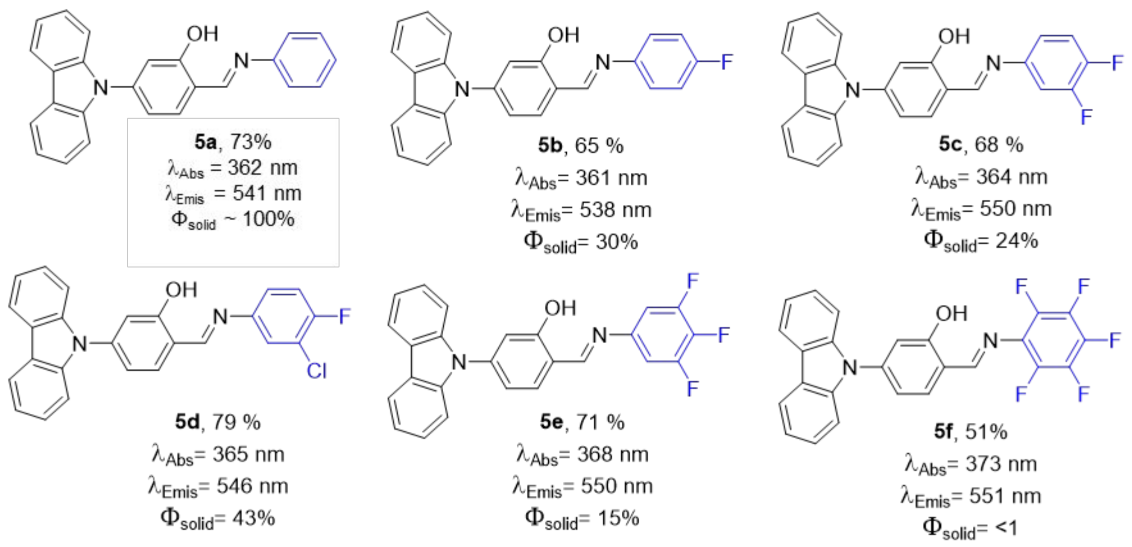

Figure S 12. Structure of synthesized compounds **5a** – **5h** with their λ_{abs} - λ_{emis} and the quantum yield in the solid state.

a. **Solution Steady-State absorption and emission in different solvents.**

Table S 3. Complete values of λ_{abs} - λ_{emis} obtained in different solvents.

	**5a**				**5b**				**5c**				**5d**			
	λ_{Abs}	λ_{Emis}	**Stokes shift**		λ_{Abs}	λ_{Emis}	**Stokes shift**		λ_{Abs}	λ_{Emis}	**Stokes shift**		λ_{Abs}	λ_{Emis}	**Stokes shift**	
			**cm^{-1}**	**nm**			**cm^{-1}**	**nm**			**cm^{-1}**	**nm**			**cm^{-1}**	**nm**
**Cyclohexane**	364	548	9224	184	366	554	9272	188	372	552	8766	180	360	552	9662	192
**Hexane**	363	543	9132	180	365	545	9049	180	370	550	8845	180	371	555	8936	184
**Toluene**	366	550	9141	184	367	553	9165	186	372	554	8831	182	372	556	8896	184
**Dioxane**	361	411	3370	50	362	410	3234	48	367	411	2917	44	367	411	2917	44
**THF**	362	541	9140	179	361	538	9113	177	364	550	9291	186	365	546	9082	181
**AcOEt**	357	543	9595	186	358	536	9276	178	362	530	8756	168	362	546	9309	184
**Chloroform**	363	541	9064	178	365	525	8350	160	369	529	8197	160	370	542	8577	172
**DCM**	360	520	8547	160	362	522	8467	160	366	528	8383	162	367	527	8273	160
**Acetone**	356	534	9363	178	356	546	9775	190	360	540	9259	180	360	538	9190	178
**DMF**	358	546	9618	188	359	521	8661	162	362	524	8540	162	362	540	9106	178
**DMSO**	359	517	8513	158	359	507	8131	148	362	506	7861	144	362	510	8016	148
**ACN**	354	544	9866	190	355	543	9753	188	359	545	9507	186	359	543	9439	184
**MeOH**	356	534	9363	178	357	531	9179	174	361	543	9285	182	361	553	9618	192
**QY % (solid-state)**	>100				30				24				43			

	5e				5f			
	λ_{Abs}	λ_{Emis}	Stokes shift		λ_{Abs}	λ_{Emis}	Stokes shift	
			cm^{-1}	nm			cm^{-1}	nm
**Cyclohexane**	376	550	8414	174	381	556	8261	175
**Hexane**	375	551	8518	176	384	556	8056	172
**Toluene**	377	557	8572	180	385	559	8085	174
**Dioxane**	371	415	2858	44	377	423	2885	46
**THF**	368	550	8992	182	373	551	8661	178
**AcOEt**	365	553	9314	188	372	556	8896	184
**Chloroform**	374	546	8423	172	381	555	8229	174
**DCM**	370	536	8370	166	377	543	8109	166
**Acetone**	363	549	9333	186	369	557	9147	188
**DMF**	365	543	8981	178	368	550	8992	182
**DMSO**	364	492	7147	128	366	508	7637	142
**ACN**	362	556	9639	194	368	550	8992	182
**MeOH**	362	558	9703	196	369	551	8951	182
**QY % (solid-state)**	15				< 1			

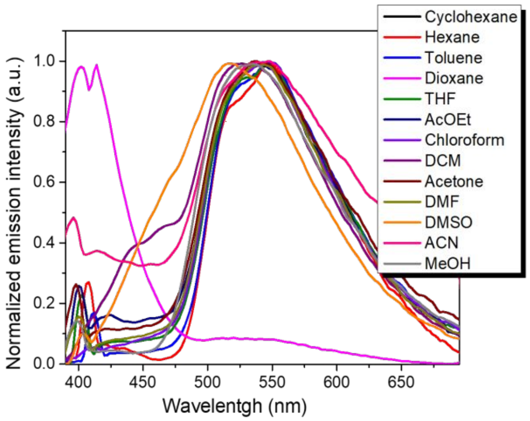

Figure S 13. Normalized emission spectra of compound **5a** in different solvents.

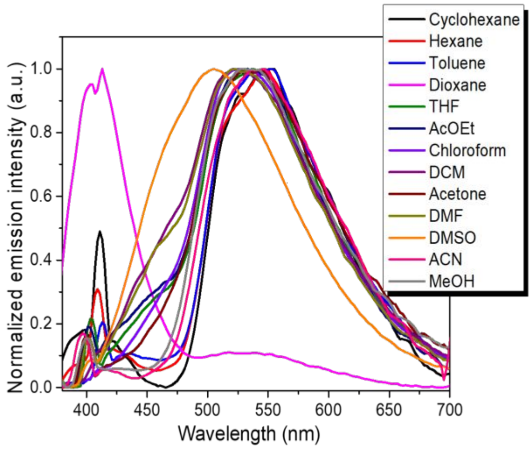

Figure S 14. Normalized emission spectra of compound **5b** in different solvents.

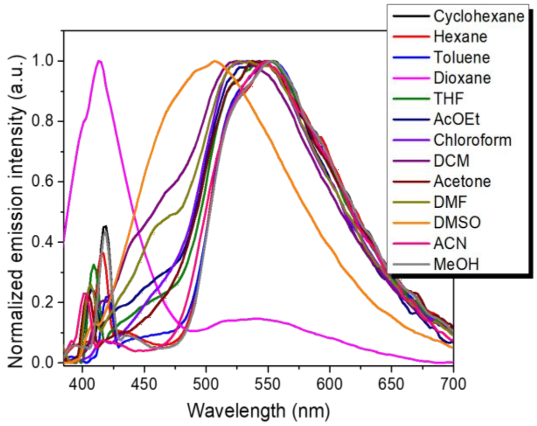

Figure S 15. Normalized emission spectra of compound **5c** in different solvents.

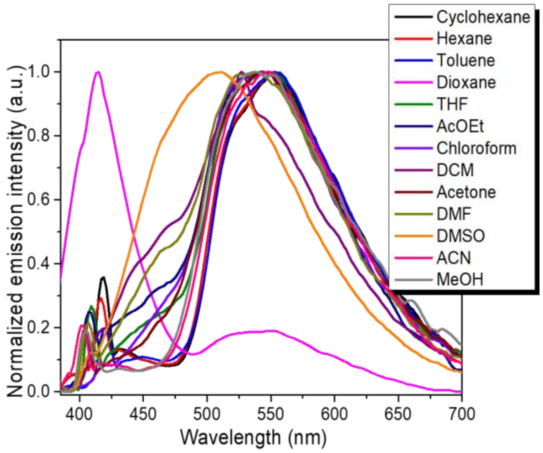

Figure S 16. Normalized emission spectra of compound **5d** in different solvents.

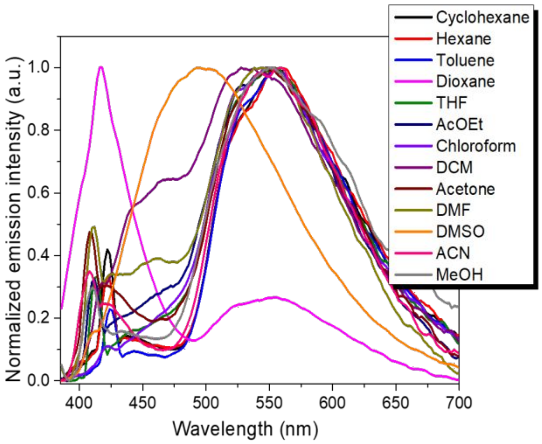

Figure S 17. Normalized emission spectra of compound **5e** in different solvents.

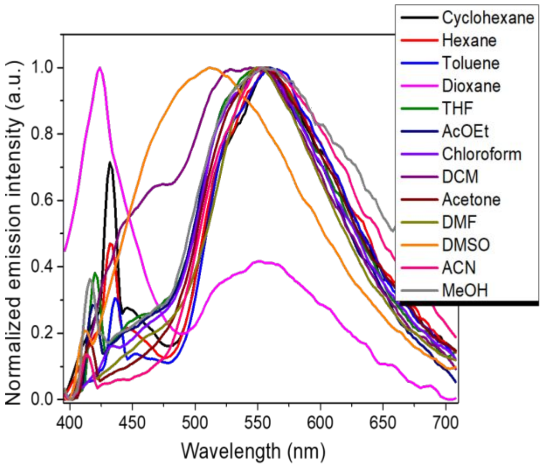

Figure S 18. Normalized emission spectra of compound **5f** in different solvents.

b. Solution Steady-State absorption and emission in different H_2O:THF mixtures

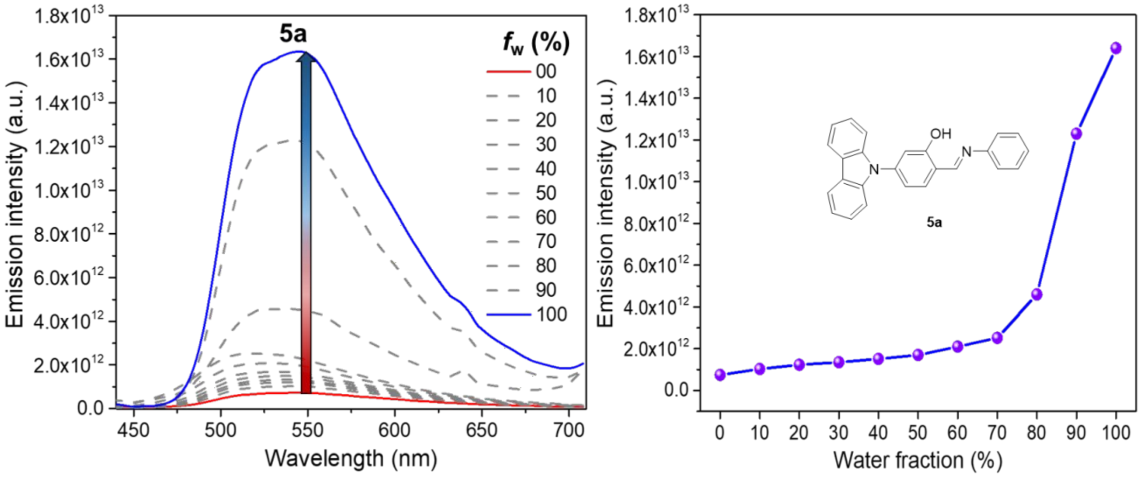

Figure S 19. Relative emission spectra (left) of compound **5a** in different mixtures THF:H_2O and change in intensity at maximum peak (right).

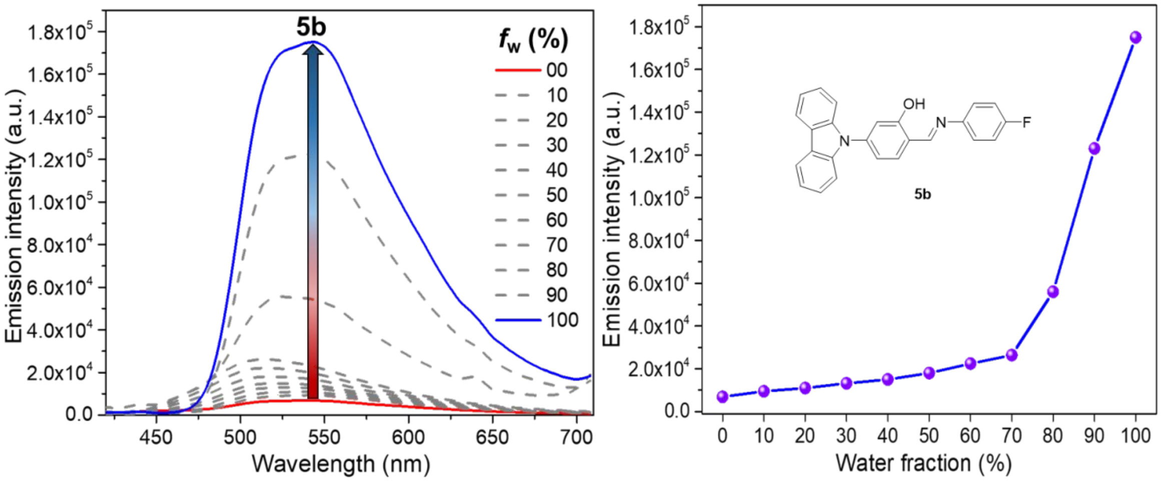

Figure S 20. Relative emission spectra (left) of compound **5b** in different mixtures THF:H_2O and change in intensity at maximum peak (right).

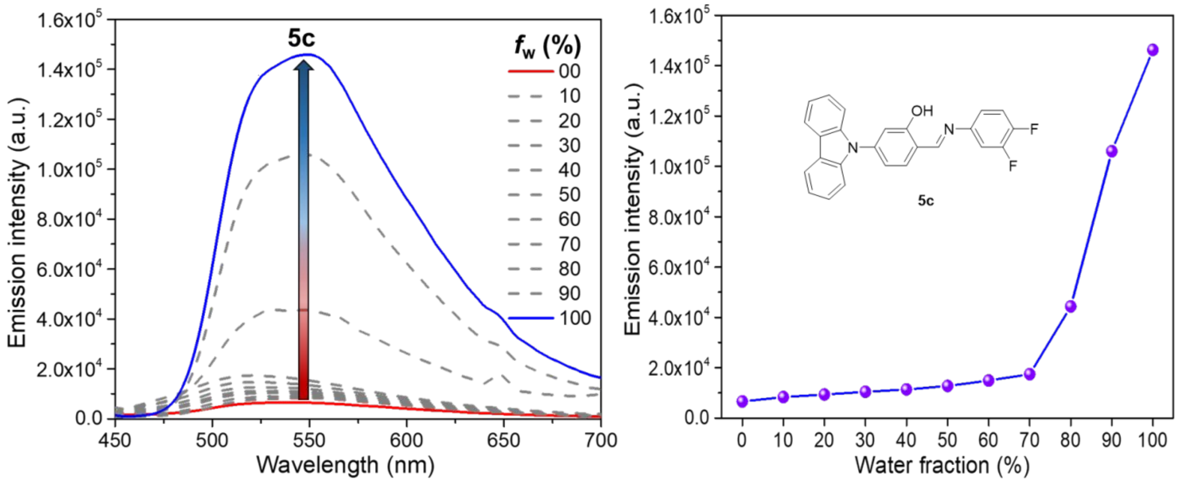

Figure S 21. Relative emission spectra (left) of compound **5c** in different mixtures THF:H_2O and change in intensity at maximum peak (right).

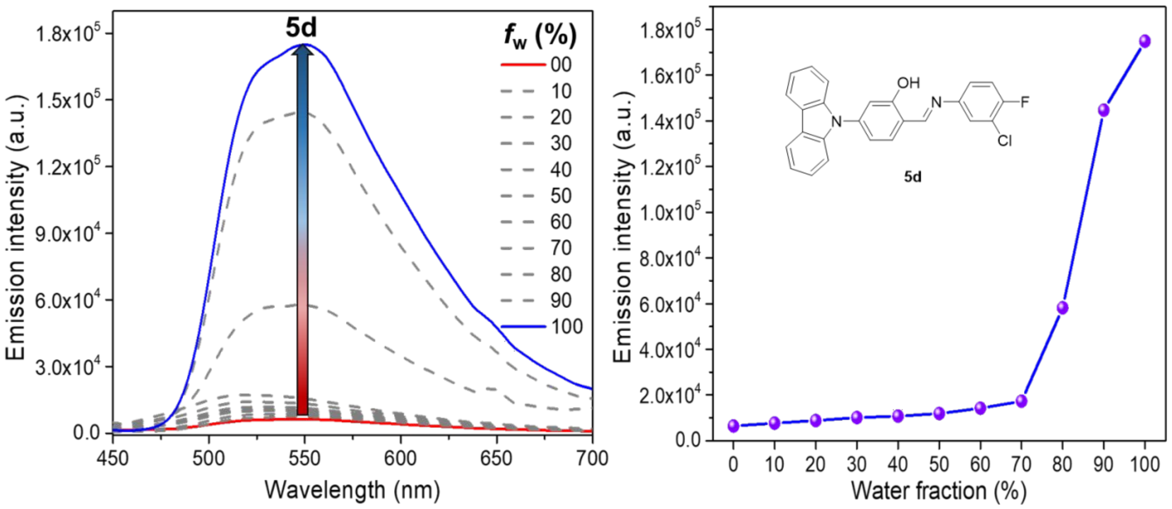

Figure S 22. Relative emission spectra (left) of compound **5d** in different mixtures THF:H_2O and change in intensity at maximum peak (right).

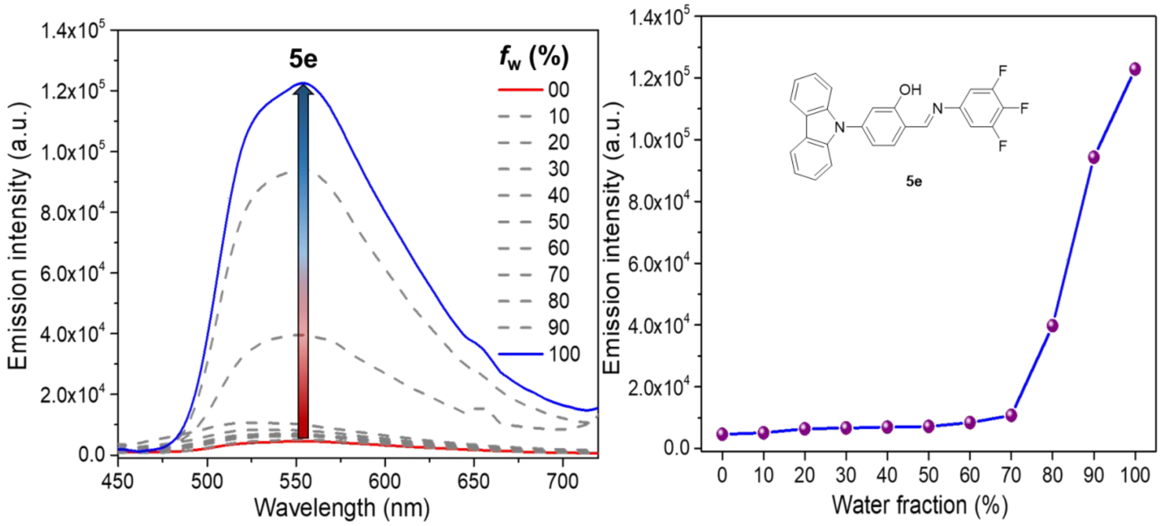

Figure S 23. Relative emission spectra (left) of compound **5e** in different mixtures THF:H_2O and change in intensity at maximum peak (right).

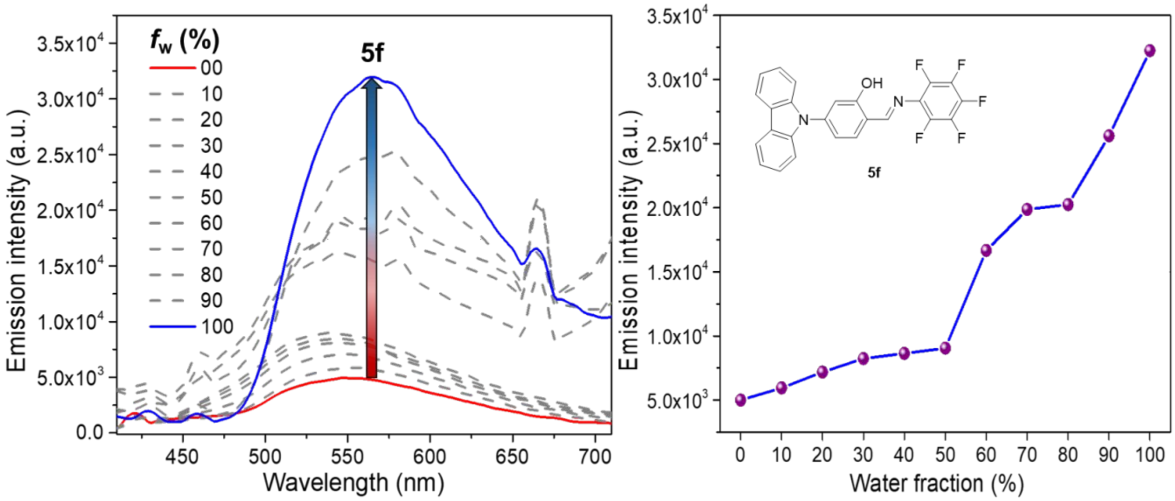

Figure S 24. Relative emission spectra (left) of compound **5f** in different mixtures THF:H_2O and change in intensity at maximum peak (right).

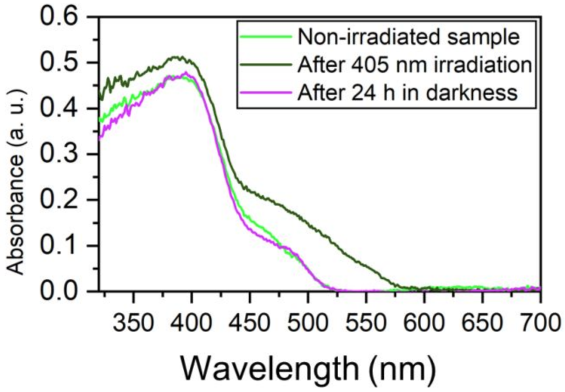

Figure S 25. UV-Vis spectra of **5e** photoisomerization
